# Multi-modal image analysis for large-scale cancer tissue studies within IMMUcan

**DOI:** 10.1016/j.crmeth.2025.101170

**Published:** 2025-09-09

**Authors:** Nils Eling, Julien Dorier, Sylvie Rusakiewicz, Robin Liechti, Preethi Devanand, Michelle Daniel, Jonas Windhager, Bruno Palau Fernandez, Sophie Déglise, Lucie Despland, Abdelkader Benyagoub, Marcin Możejko, Dawid Uchal, Ewa Szczurek, Alexander Loboda, Daaf Sandkuijl, Nikesh Parsotam, Henoch S. Hong, Marie Morfouace, Nicolas Guex, George Coukos, Bernd Bodenmiller, Stephanie Tissot, Daniel Schulz

**Affiliations:** 1 Department of Quantitative Biomedicine, University of Zurich, Zurich, Switzerland; 2 Institute of Molecular Health Sciences, ETH Zurich, Zurich, Switzerland; 3 Bioinformatics Competence Center, University of Lausanne, 1015 Lausanne, Switzerland; 4 Department of Oncology, Centre Hospitalier Universitaire Vaudois, Lausanne, Switzerland; 5 Ludwig Institute for Cancer Research, Lausanne Branch, Lausanne, Switzerland; 6 Immune Landscape Laboratory, Centre Thérapies Expérimentales (CTE), Centre Hospitalier Universitaire Vaudois, Lausanne, Switzerland; 7 Vital-IT Group, SIB Swiss Institute of Bioinformatics, Lausanne, Switzerland; 8 Life Science Zurich Graduate School, ETH Zurich and University of Zurich, Zurich, Switzerland; 9 Faculty of Mathematics, Informatics and Mechanics, University of Warsaw, Warsaw, Poland; 10 Institute of AI for Health, Helmholtz Zentrum Munich, Munich, Germany; 11 Standard BioTools Canada, Inc., Markham, ON, Canada; 12 Merck KGaA, Darmstadt, Germany; 13 EORTC HQ, Avenue E. Mounier 83/11, 1200 Brussels, Belgium; 14 Institut Gustave Roussy, Villejuif, France

**Keywords:** multiplexed imaging, imaging mass cytometry, spatial proteomics, tumor microenvironment, workflow, cancer, IMMUcan consortium, image analysis, cell segmentation, single-cell analysis

## Abstract

In cancer research, multiplexed imaging allows detailed characterization of the tumor microenvironment (TME) and its link to patient prognosis. The integrated immunoprofiling of large adaptive cancer patient cohorts (IMMUcan) consortium collects multi-modal imaging data from thousands of patients with cancer to perform broad molecular and cellular spatial profiling. Here, we describe and compare two workflows for multiplexed immunofluorescence (mIF) and imaging mass cytometry (IMC) developed within IMMUcan to enable the generation of standardized data for cancer tissue analysis. The IFQuant software supports web-based, user-friendly, and reproducible analysis of mIF data. High sample throughput for IMC is achieved by optimizing experimental protocols, developing a robotic arm for automated slide loading, and classification-based cell typing. Using our manually labeled single-cell data, we show that tree-based methods outperform other cell-phenotyping tools. These pipelines form the basis for multiplexed image analysis within IMMUcan, and we summarize our learnings from 5 years of development and optimization.

## Highlights

• Standardized workflows enabling large-scale multiplexed cancer tissue profiling
• *IFQuant* open-source software for multiplexed immunofluorescence analysis
• Tree-based machine learning outperforms other tools in IMC cell-type classification
• High consistency between mIF and IMC cell types

## Motivation

Multiplexed imaging is increasingly used to study tissue architecture in health and disease. To investigate the cancer tumor microenvironment, typically either tissue micro-arrays or small patient cohorts are used to collect and process data. However, studies performed over the course of years, collecting data from thousands of samples, are rare and require specialized workflows to ensure sample throughput and reproducibility for data production and processing. Here, we present two such workflows for multiplexed immunofluorescence and imaging mass cytometry of cancer tissues, which are applied to a total of roughly 10,000 samples from more than 2,500 patients over 6 years.

## Graphical abstract

**Figure:**
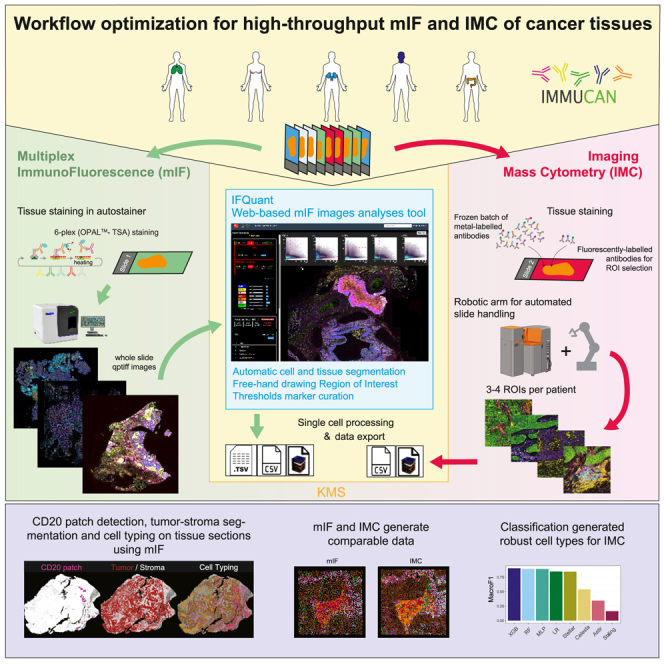

## Teaser

Eling and Dorier et al. present standardized workflows for multiplexed immunofluorescence and imaging mass cytometry, enabling large-scale profiling of thousands of cancer tissues in the pan-European IMMUcan consortium. The authors demonstrate high cross-technology consistency and release open-source tools and datasets to support community-wide immune landscape studies.

## Introduction

The emergence of successful immunotherapy has revolutionized cancer treatment in recent years. However, good prognostic markers for immune response in patients are still lacking, and some patients even acquire resistance.^1^^,^^2^ The tumor microenvironment (TME) is composed of tumor cells, immune cells, fibroblasts, and endothelial cells and has pivotal anti- and pro-tumorigenic functions,^3^^,^^4^ and it is commonly accepted that the context of tissue architecture is crucial. Therefore, to reveal the immune cell content of the TME, technologies have been developed to spatially characterize cells in tissue sections.^5^ Numerous studies applied multiplexed imaging to investigate the TME and identified signatures of poor or good survival or those that are predictive of treatment.^6^^,^^7^^,^^8^^,^^9^^,^^10^^,^^11^ However, efforts to profile thousands of cancer patients with spatially resolved single-cell technologies and molecular data do not exist. To systematically profile the variation of the TME and to characterize biomarkers for diverse treatments, the integrated immunoprofiling of large adaptive cancer patient cohorts (IMMUcan) consortium acquires molecular and cellular profiles of over 2,500 patients across five cancer types up to 2026.^12^ By integrating single-cell data obtained from multiplexed imaging technologies with bulk and whole-exome sequencing data and their associated clinical metadata, factors for improved patient stratification or treatment prediction can be unveiled. Within IMMUcan, formalin-fixed and paraffin-embedded (FFPE) tumor samples are processed for molecular and cellular profiling. To study single cells within their spatial tissue context, mass spectrometry-based technologies such as imaging mass cytometry (IMC)^13^ (Standard BioTools) and multiplexed immunofluorescence^14^ (mIF) are established technologies. Both technologies produce complementary readouts capturing the spatial distribution of dozens of biomolecules, including proteins. While mIF allows the detection of 6–7 fluorescence readouts across the whole cancer tissue, IMC captures ∼40 proteins in smaller (∼1 mm^2^) regions of the tissue. The IMMUcan consortium generates images using both IMC and mIF to thoroughly profile the TME across different tumor types and thousands of patients. This endeavor raises a number of challenges. First, scalable and reproducible software tools need to be provided to analyze mIF images with up to millions of cells per image. Second, the robust and informative selection of regions of interest (ROIs) for IMC^15^^,^^16^ needs to be performed. Third, an ongoing challenge is the precise detection of cell types across images, patients, and cancer types.^17^^,^^18^^,^^19^^,^^20^^,^^21^^,^^22^^,^^23^ Finally, a key challenge for a large-scale project such as IMMUcan is to ensure the reproducible processing and analysis of thousands of individual samples over a period of 6 years.

Here, we present, and put into perspective, the two independently developed workflows and computational tools for mIF and IMC data generation and processing within IMMUcan. We perform a quantitative comparison of the results of the two workflows and show high correlation and spatial co-localization for all major cell types between mIF and IMC. Alongside this, we provide a ground-truth dataset of IMC images from 179 patients with manually annotated labels of 14 cell types for further method development. Our large-scale data acquisition, handling, and analysis approaches result in the accurate application of both mIF and IMC to large patient cohorts and ensure the high quality and comparability of the data for downstream analysis and characterization of the TME.

## Results

### Highly multiplexed imaging for broad immune profiling of cancer samples

**Figure 1:**
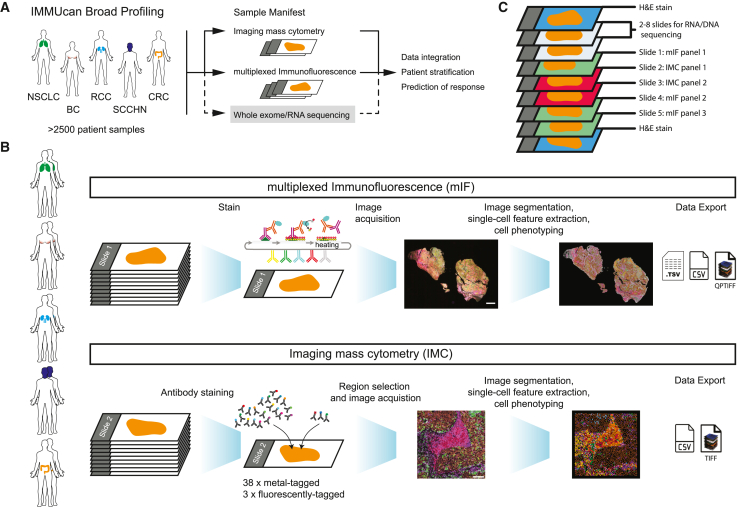
Multiplexed imaging for broad immune profiling of cancer tissues (A) The IMMUcan consortium collects and analyzes cancer tissues of more than 2,500 patients diagnosed with one of five cancer types: breast cancer (BC), renal cell carcinoma (RCC), squamous cell carcinoma of head and neck (SCCHN), colorectal carcinoma (CRC), or lung cancer (NSCLC). (B) Ten samples across all five cancer indications were selected to demonstrate the data generation and analysis workflows for IMC and mIF. One section of each patient tumor was stained with 6 antibodies plus DAPI using the PhenoImager HT for whole-slide fluorescence detection. Image analysis included cell segmentation, feature extraction, and cell phenotyping. The next consecutive slide of each patient was stained with 38 metal-tagged antibodies and 3 fluorescent-labeled antibodies. Processing included segmentation, feature extraction, and cell phenotyping, followed by standardized data output for the consortium. Scale bars: 2 mm for mIF and 100 µm for IMC. (C) FFPE blocks from eligible patients were collected and sectioned as shown above. Typically, consecutive sections can be rotated, and their absolute position on the slides varies. Depending on the size of the tumor, a variable number of sections were cut for RNA sequencing (RNA-seq) and whole-exome sequencing (WES). Scale bars: 2 mm for mIF and 100 μm for IMC. Image composites of epithelial cell markers (cytokeratin [mIF] and E-cadherin [IMC], brown), CD20 (B cells, red), CD3 (T cells, magenta), and CD163 (macrophages, green) are shown.

The broad profiling team of the IMMUcan project collects and analyzes imaging data from five cancer types: breast cancer (BC), renal cell carcinoma (RCC), squamous cell carcinoma of head and neck (SCCHN), colorectal carcinoma (CRC), and non-small cell lung cancer (NSCLC) (Figure 1A; STAR Methods).

We illustrate the mIF and IMC data generation and evaluation pipelines developed within the IMMUcan project using 10 samples that cover each of the five cancer indications (Figure 1B). For each patient, typically three tissue sections are stained for mIF with 6-plex antibody panels + DAPI using the tyramide signal amplification (TSA) technology,^14^ and two consecutive tissue sections stained with two 40-plex antibody panels for IMC (Figures 1C; Table S1). Here, we demonstrate the workflows using tissue sections of the first mIF panel and the consecutive first IMC panel. IMC is limited in throughput and requires the selection of ROIs. An optimized, IF/IMC co-stain allowed us to perform guided ROI selection (STAR Methods). The computational workflows for mIF and IMC data analysis are similar and include sample tracking, image processing and segmentation, single-cell feature extraction, and cell phenotyping, as well as tissue structure detection and quality control (QC) (Figure 1B). Features are exported to CSV files along with a QC matrix and QPTIFF images for mIF and TIFF images for IMC.

### mIF experimental and quantification workflow

**Figure 2:**
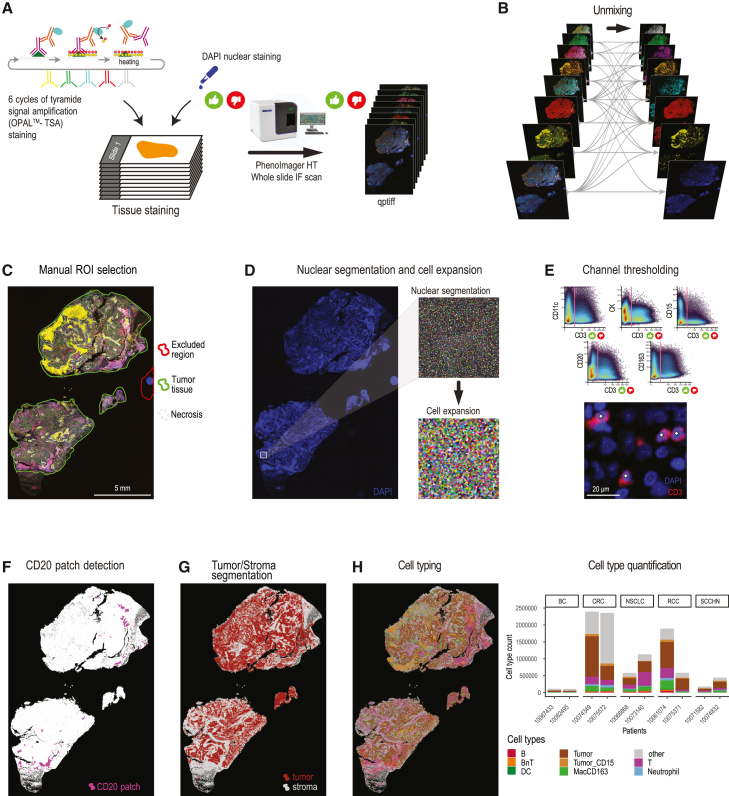
Overview of mIF experimental and IFQuant image analysis workflow (A) Tissue staining was performed using 6 cycles of tyramide signal amplification with specific antibodies and DAPI for nuclear staining. Slides were scanned using the PhenoImager HT. (B and C) Quantification workflow: images were spectrally unmixed (B) and annotated (C) using IFQuant. (D) Single cells were defined using DAPI-based nuclear segmentation followed by nuclear mask expansion for single-cell detection. (E) Channel-specific thresholding is performed manually to identify marker-positive cells. Scale bar: 20 µm (F) CD20 patches are detected as proxy for TLSs, based on CD20 signal (shown in pink). (G) Areas of predominant tumor or non-tumor cells are detected and annotated automatically (tumor depicted in red and stroma in gray). (H) Identified cell types based on marker positivity are shown in the image (left) together with a quantification of total cell counts per sample and cell type (right). Little thumbs up/down icons indicate quality control checks, which are logged in the software (STAR Methods). Scale bar in (C): 5 mm; (D), (F), (G), and (H) shown at identical magnification.

To profile the spatial distribution of major immune phenotypes across whole-tissue slides, we optimized a 6-plex panel of antibodies (CD3, CD11c, CD15, CD20, CD163, and cytokeratin [CK]; Table S1) supplemented with DAPI for nuclear staining. Initial images were acquired using the Ventana automated stainer and the TSA OPAL technology^24^ combined with the PhenoImager HT (Figure 2A; STAR Methods). For large-scale processing, a set of critical challenges needed to be solved. Among those were sample tracking, standardized analysis, data normalization, output, and report generation. Initially, commercial software was used for mIF analysis. However, it was not feasible to robustly track samples during different stages of processing, making data corrections, parameter extractions, and data version bumps a critical challenge. Therefore, we designed and developed IFQuant, a web-based tool to analyze multiplexed mIF data, fully integrated in the laboratory integration management system (LIMS), with a specific focus on sample tracking, reproducibility, scalability, and user friendliness. Once registered, samples can obtain multiple IDs for barcode generation, are tracked through processes with storage of experimental or analytical parameters within the LIMS, and data output is transferred automatically to specific server destinations. A standalone version of the software is available as a Docker container, and a detailed description and screencast of the IFQuant analysis workflow can be found in Methods S1 and Video S1.

Video S1. Screencast of the *IFQuant* web interface usage, related to STAR Methods

IFQuant directly accesses QPTIFF image files from the LIMS and performs multi-channel signal unmixing (Figure 2B; STAR Methods). IFQuant provides an integrated web interface to facilitate manual analysis steps. The operator can use a free-hand drawing tool to assign manual annotation masks to the images to either filter unwanted regions (e.g., out of focus or bad tissue quality) or label specific tissue regions (e.g., tumor, necrosis, healthy tissue, or adipose tissue, Figure 2C). Nuclear segmentation is performed automatically based on the DAPI signal, and single cells are defined based on an expansion of the nuclear mask (Figure 2D; STAR Methods). Of note, IFQuant uses expansion for whole-cell detection since the mIF panels cannot cover cytoplasmic or membranous markers for all cell types.

Since the start of the IMMUcan project, a number of segmentation methods and image analysis tools have been published.^25^^,^^26^^,^^27^^,^^28^^,^^29^^,^^30^ To investigate the performance of IFQuant, we compared the cell segmentation of six tools with IFQuant (Figure S1). We found that DeepCell for nuclear segmentation and UnMICST with standard settings performed poorly in our hands and missed many nuclei (Figures S1A and S1B). DeepCell for whole-cell detection (Mesmer), on the other hand, detected the largest number of nuclei, of which many were not detected with other methods (Figure S1B). IFQuant detected more nuclei than cellpose or InstanSeg and fewer nuclei than StarDist or Mesmer. Our analysis also revealed that IFQuant had a slight tendency to merge nuclei compared to cellpose, InstanSeg, and StarDist (Figure S1C). In summary, IFQuant performed similarly to state-of-the-art methods for nuclei segmentation of fluorescence data that progressively became available during the lifespan of the project. Nonetheless, we observed remarkable deviation across the results of the segmentation tools, implying that quality control of any of the tools is required.

Due to sample-to-sample variations in tissue quality and potential minor differences in pretreatment, fluorescence-based marker signal intensity ranges can vary. Therefore, the operator utilizes the IFQuant web interface to refine automatically set thresholds for each marker to extract positive and negative cells, which can simultaneously be visually inspected for quality control. This adjustment is facilitated by a high-resolution image viewer that presents cell overlays and density scatterplots of marker intensities (Figure 2E). IFQuant automatically detects B cell patches as proxies for tertiary lymphoid structures (TLSs) based on the threshold for CD20 positivity (Figure 2F; STAR Methods). IFQuant automatically detects areas consisting mostly of tumor cells (CK-positive cells) and areas consisting mostly of stromal and immune cells (CK-negative cells) and generates a tumor-stroma mask thereof (Figure 2G; STAR Methods). During post-processing, the mIF marker panel allows annotation of cells as B cells, BnT cells (cell positive for CD20 and CD3), T cells, dendritic cells (DCs), neutrophils, macrophages (MacCD163), “other,” and epithelial tumor cells (Figure 2H) using our phenotype key, linking cellular phenotype and marker positivity (Table S1). IFQuant outputs one standardized tabular file (CSV file) containing marker expression, binary marker positivity, the spatial *xy* location for each cell, and information regarding annotations (e.g., in-tumor, necrosis, etc.) for downstream computational analysis.

We compared the capabilities of IFQuant to those of commercial and non-commercial software with respect to important software criteria for a project such as IMMUcan (Table 1). This comparison shows that currently, no software other than IFQuant offers out-of-the-box properties as required for IMMUcan. Software such as QuPath,^31^ Visiopharm, or InForm are not readily integrable into the LIMS and also partially lack channel deconvolution. MCMICRO,^32^ on the other hand, could be modified to allow integration into the LIMS but does not allow QPTIFF analysis and would need adaptation for image viewing.

**Table 1:** Comparison of different mIF analysis software

	IFQuant	InForm	QuPath	Visiopharm	MCMICRO
**Software**					

Proprietary	no (open source)	yes (AKOYA)	no (open source)	yes	no (open source)
Available at IMMUcan project start (March 2019)	developed for this project	yes	yes (Bankhead et al.^31^)	yes	no (Schapiro et al.^32^)
Integration into LIMS	yes	no	no	no	yes (development needed)

**Image processing**					

Analyses of QPTIFF image	yes	yes	yes	yes	no, need conversion
Deconvolution of 7-plex QPTIFF	yes	yes	no	no	no
Cell segmentation	fixed parameters for PhenoImager (QPTIFF)	parameter setting required	optional parameter fine-tuning	optional parameter fine-tuning	optional parameter fine-tuning
Tissue segmentation based on tumor marker	automatic (manual curation possible)	training and classification	training and classification	training and classification	optional parameter fine-tuning
Marker phenotyping	assisted manual thresholding	training and classification	training and classification	training and classification	unsupervised clustering or manual gating
Server version availalble	yes	no	yes	yes	yes

**Interface visualization**					

Visualization of image	whole image	small subset	whole image	whole image	no, possible integration with other tools
Region of interest definition (free-hand drawing)	yes	no	yes	yes	yes if integrated with other tools (napari, Omero)

**Output**					

Single-cell data table	yes	yes	yes	yes	yes
Quality control report	yes	no	no	no	no

In conclusion, the IFQuant software streamlines the management and analysis of mIF imaging data, requiring typically 10–15 min of operator time per tissue section and up to 30 min for very large or damaged tissues, and delivers a comprehensive and standardized output of spatially resolved single-cell data for subsequent analysis.

### Frozen antibody mixes enable IMC measurement comparison over time

**Figure 3:**
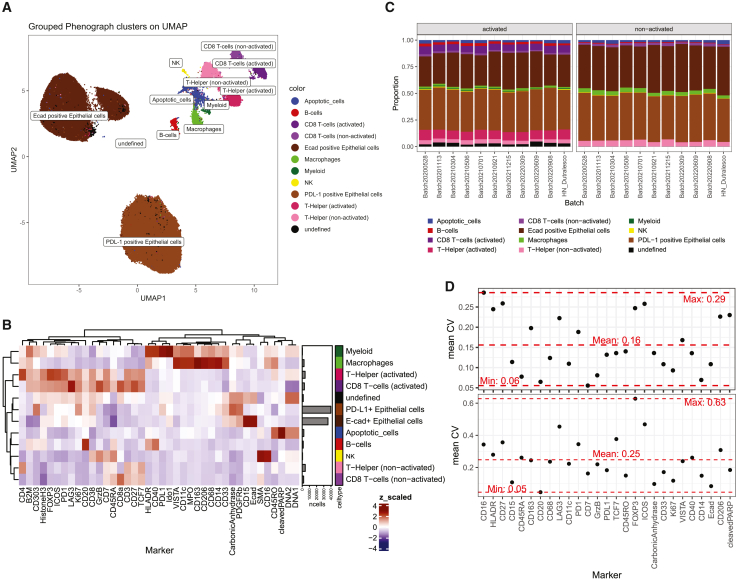
IMC control cell pellets identify variation over time (A) Uniform manifold approximation and projection (UMAP) of all cells acquired from sections of cell pellets between May 2020 and September 2022. Cells are colored and labeled according to cell type. (B) Heatmap of *Z* score-scaled marker expression averaged per cell type. Barplots show the abundance of each cell type in the data. (C) Barplot showing the proportions of cell types of the cell pellets for each sample batch acquired over 2 years. Fractions are colored according to cell type. (D) For each marker expressed in a certain cell type, the mean coefficient of variation (CV) over all time points (sample batches) is shown (black points) on top for activated and on the bottom for non-activated cell pellets. The horizontal red dashed lines indicate the maximum, minimum, and mean observed CVs across all markers.

Individual sample stainings can be a source of batch effects. To minimize such effects throughout the IMMUcan project, we worked with large antibody mixes sufficient to stain 500 patient samples (STAR Methods).^33^^,^^34^ For each set of 19 patient samples, we stained and acquired one slide containing two control cell pellets. One cell pellet contained a mix of an epithelial cell line and non-activated peripheral blood mononuclear cells (PBMCs). The other cell pellet contained PBMCs activated with phytohemagglutinin (PHA) and epithelial cells cultured with interferon (IFN)-γ, thereby upregulating the expression of PD-L1 (STAR Methods). To investigate the reproducibility in cell-type detection and marker expression over time, the images acquired from control cell pellets over the course of more than 2 years were processed, and single cells were clustered and annotated to identify cell types (Figure 3A; STAR Methods). Epithelial cells were separated based on IFN-γ treatment, while PBMCs were separated by cell type and activation (Figures 3A, 3B, and S2A). Additionally, we observed a small batch effect based on the antibody staining mix visible on non-activated epithelial cells (Figures S2B and S2C). The fraction of detected cell types in the cell pellets showed mean coefficients of variation of 15% and 25% for cell types in activated and non-activated cell pellets, respectively (Figures 3C and S2D). We compared the expression of relevant markers for cell types across batches and similarly found mean coefficients of variation of 16% and 25% for activated and non-activated cell pellet samples, respectively (Figure 3D). The variation in staining was highly correlated with the expression of individual markers (R^2^ = 0.66), implying that lower signal intensities require larger statistical power for differential detection (Figure S2E). The variation observed across cell pellets for 2 years suggests that the data from the cancer images acquired during the IMMUcan project will be comparable. The remaining staining variation, together with patient-to-patient variation, needs to be accounted for in statistical testing to detect consistent changes between patient groups.

### Optimized IMC/IF co-stain for region selection and high throughput of whole-slide samples

**Figure 4:**
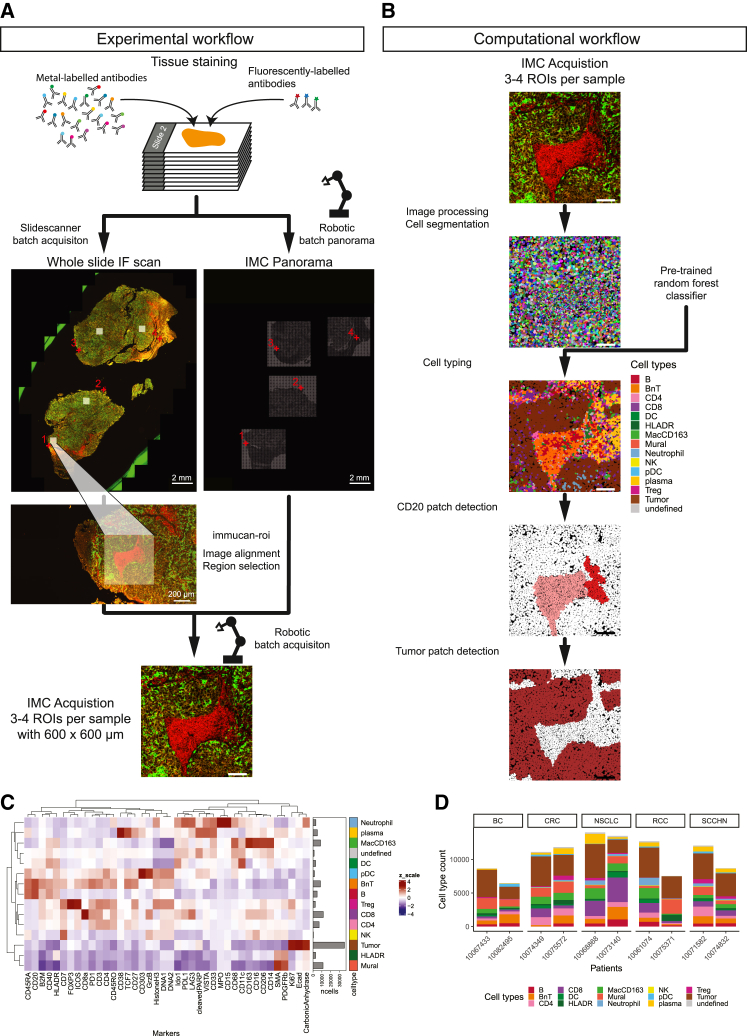
IMC workflow for high-throughput acquisition and analysis of whole-tissue slides (A) Tissue sections are stained using metal-labeled and fluorophore-labeled antibodies. After staining and drying, slides are immediately scanned overnight using a slide scanner, and subsequently, a slide loader is used to generate panoramas for IMC. The fluorescence whole-slide scan and panoramas are then used to align the two modalities. Once aligned, ROIs are selected on the IF image and automatically transformed into IMC coordinates. The slide loader is used to acquire the selected ROIs in batches of up to 40 slides, with a variable number of ROIs per slide. (B) The computational workflow encompasses pre-processing and single-cell segmentation. Cell phenotyping is performed with a pre-trained classifier, and patches of B cells and tumor cells are detected. (C) Heatmap showing the *Z* score-scaled mean marker expression in cell types classified in the 10 samples used in this study. Barplots indicate the abundance of each cell type in the data. (D) Barplots showing the cell-type counts for two samples of each cancer type. Scale bars: 2 mm (A) or 200 µm (A) as indicated. Scale bars without without size declaration: 100 µm (A and B).

To stain, measure, and analyze thousands of tissue sections using IMC in a reproducible and scalable fashion over 6 years, we optimized an experimental and computational workflow (Figure 4). Hyperion+ IMC systems acquire images at about 1 mm^2^ per hour and therefore require the selection of ROIs for multiplexed profiling of whole-slide tissues. Of note, the latest machine from Standard BioTools, the XTi released in 2024, is faster and offers a new feature for region selection, for which we highlight possible implications in the discussion. Hyperion and Hyperion+ IMC systems only allow the acquisition of bright-field overview images (panoramas) for ROI selection. To guide region selection, whole-slide IF staining and imaging prior to metal-labeled antibody staining have been used in the past for supervised ROI selection.^35^^,^^36^^,^^37^ However, these multi-step staining protocols are too laborious for the throughput of the IMMUcan project. Therefore, we evaluated the possibility of simultaneously co-staining primary, fluorescent-labeled antibodies with metal-tagged antibodies and detecting the fluorescent signal in dried tissues as required for IMC (Figure 4A). We selected antibodies against CD45, CD163, and pan-CK to identify immune cells, macrophages, and epithelial cells in an mIF co-stain. Fluorescence images after tissue drying were of good quality (Figures S3A and S3B), and we did not observe a decrease in signal intensity within the first 48 h after tissue drying (Figure S3C). Importantly, the joint use of IMC and IF antibodies against the same epitope could potentially reduce the signal of either of the antibodies. We therefore carefully identified fluorescent-labeled antibody clones against CD45 and CD163 that maintained the signal from metal-labeled antibodies targeting CD45RA, CD45RO, and CD163 (STAR Methods). These fluorescent-labeled antibodies were added to the metal-labeled antibody mix for standard staining of IMMUcan samples (Table S1; Figure 4A).

### Automated slide loading and region selection for IMC

The Hyperion+ IMC system supports the measurement of one slide at a time. To increase the throughput and scale to the measurement of thousands of slides, we collaborated with Standard BioTools to develop a slide loader for batch processing of microscopy slides. The Hyperion Imaging System and its software were customized to allow the installation of a robotic arm with a slide hotel carrying up to 40 slides. A commercial solution of the slide loader is now available for the XTi generation of IMC machines, substantially increasing sample throughput. Nonetheless, IMC with single-cell resolution is not suited for whole-slide imaging, and ROIs have to be defined. We decided to use a fluorescence-guided approach, optimized for efficiency, albeit requiring manual intervention. For this, we made use of the co-stained fluorescence data from the same tissue section and selected ROIs containing a mix of tumor and immune cells using a custom plugin for the napari image viewer^38^ (Figure 4A; STAR Methods). Using the slide loader, batches of up to 40 slides could be processed sequentially without intervention, thereby enabling large single-slide studies with IMC.

### Downstream analysis of IMC images and cell phenotyping

We applied a computational workflow for data analysis that includes image pre-processing, cell segmentation, feature extraction, cell phenotyping, and spatial analysis for IMMUcan. For raw data processing, cell segmentation, and feature extraction, we use the steinbock toolkit (Figure 4B; STAR Methods). Most single-cell analysis steps follow our standard workflow (available at https://bodenmillergroup.github.io/IMCDataAnalysis/).^39^ To robustly define cell types across shipment batches for the IMMUcan project, we performed random-forest-based classification (STAR Methods). Within the consortium, we defined the major cell types, given the antibody panel (Figure S4A). We selected 179 images from individual patients from data acquired over 2 years and manually annotated more than 340,000 cells. Images were selected to represent different sample batches and cancer indications, and manual gating was performed for all cell types following a general scheme (Methods S2). Gating was performed on the single-cell data, and gates were inspected on the images (Figure S4B). We trained a random forest classifier on 80% of the annotated images while providing the cancer indication as a covariate and validated the classifier on the remaining 20% of the annotated images (STAR Methods). The classifier detected most cell types with a true positive rate (TPR) greater than 0.8 and a false positive rate (FPR) smaller than 0.03 (Figure S4C) and is standardly applied to all samples stained within IMMUcan to detect the defined cell types (Figures 4C and 4D). Based on the detected cell types, we define CD20 patches and tumor patches (Figure 4B; STAR Methods). In sum, the developed computational workflow is reproducible and scalable and generates results for around 100 samples, containing 0.5–1 million cells, in around 10 h as part of standardized IMMUcan sample processing (with parallel processing on a 32-core, 128 GB RAM Ubuntu system).

### Cell-type classification benchmark of IMC data

**Figure 5:**
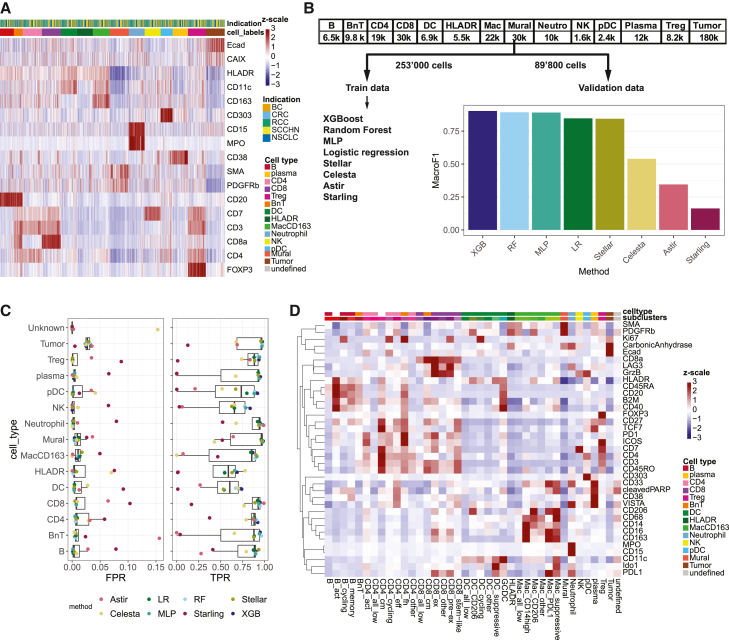
Cell-type calling and benchmarking in IMC (A) Heatmap showing the *Z* score-scaled mean marker expression per image of markers used to label cells in rows and the labeled cells in columns. (B) Overview of numbers of labeled cells per cell type (top). Labeled cells were split at a 80:20 ratio into training and test data. Different machine learning methods (left) were trained on the training data. The MacroF1 scores from applying the trained models on the test data shown on the right. (C) For each labeled cell type, the false positive rate (FPR) and the true positive rate (TPR) are color coded for each of the models used to predict cell types of the test data. (D) Heatmap showing the *Z* score-scaled mean expression per sub-cluster of markers (rows) for sub-clustered cell types (columns). B_act and CD4_cm sub-clusters originate from multiple cell types and therefore do have missing colors for cell types.

The automated identification of cell types such as lymphocytes, myeloid cells, tumor cells, and functional subsets thereof is an active area of research.^17^^,^^18^^,^^19^^,^^20^^,^^21^^,^^22^^,^^23^ The labeled IMC data are a valuable resource, which contains manually annotated, high-quality major cell types (Figure 5A) and is ideally suited to investigate the performance of state-of-the-art methods on our data. Our dataset contained a total of close to 343,000 cells with varying amounts of individual cell types (Figure 5B). We reused the 80:20 train/test split of the annotated data and trained/validated multiple machine learning as well as recently published cell-typing algorithms (Figure 5B; STAR Methods). Taking the MacroF1 score as an overall performance metric, we found that classic machine learning models like XGBoost or random forest outperformed other algorithms. Inspection of TPRs and FPRs per cell type revealed that all models showed low performance when detecting HLADR^+^ cells (negative for CD20, CD11c, and CD163), DCs, and natural killer (NK) cells (Figure 5C). Inspection of the confusion matrices revealed that Astir^22^ distributed most cells except T cells, tumor cells, and neutrophils, among numerous other cell types (Figure S5A), while Celesta^20^ tended to assign cells to an unknown class, particularly macrophages (Figures 5C and S5B). Starling was primarily developed to model true cellular phenotypes, given underlying segmentation errors.^23^ In our dataset, we observed that Starling did indeed split all BnT cells into either B or T cells, but most other cell types were also assigned to different cell types, making it perform poorly in our case (Figure S5C). Stellar,^19^ a geometric deep learning method, performed well, and most of the wrongly classified cells were assigned to tumor cells (Figure S5D). In summary, classic machine learning models such as XGBoost or random forest outperformed some of the recently published methods and seem highly suited for our task of identifying predefined cell types.

The high multiplexity of IMC offers the potential to detect more fine-grained cell types than those classified. However, the definitions of those functional subsets vary, and their detection requires manual intervention and thorough quality control. For these reasons, we decided to identify solely major cell types in the IMMUcan data in a high-throughput fashion. To showcase that sub-clustering with relevant markers for each of the high-quality major cell types can be used to reveal functional subsets of cells, we sub-clustered BnT, CD8^+^, and CD4^+^ T cells, macrophages, and DCs (Figure 5D; STAR Methods). We identified different populations of B cells (activated and memory), CD4^+^ T cells (activated, central memory, cycling, follicular helper, and effector), CD8 T cells (central memory, exhausted, pre-exhausted, and stem-like), populations of DCs (suppressive, cycling, CD206^+^, and germinal center DC), and different macrophage phenotypes (CD14^+^, CD206^+^, PDL1^+^, and suppressive). Sub-clusters such as central memory CD8^+^ and CD4^+^ T cells and follicular T helper cells typically also contained signals from CD20 since they mostly resided in proximity to B cells. We conclude that classification enables robust detection of major cell types over time, which can subsequently be sub-clustered to identify fine-grained functional states.

### Cell types and spatial features are comparable between IMC and mIF

The experimental and computational workflows for mIF and IMC newly developed within IMMUcan were designed to ensure high reproducibility and throughput, enabling the acquisition of data from thousands of patients. We next compared the results of these two pipelines to assess whether cell types and their spatial relations are comparable between the two technologies. This comparison forms the basis for downstream analysis steps within the IMMUcan project, including patient stratification and biomarker discovery based on cell types and spatial features.

We analyzed nearly 10 million cells in mIF and 100,000 cells in IMC across the 10 samples processed here. While cell numbers from IMC, which analyzes roughly equal regions of tissue, were comparable across samples (6,477–14,041 cells per sample), we observed larger variation in cell numbers in mIF, ranging from around 100,000 cells in biopsies to more than 2 million cells in large surgical specimens. For mIF, cell phenotyping was performed by manual thresholding of marker intensities and annotation of 8 unique phenotypes (Figure 2H). Cell phenotyping in IMC was performed by classification and resulted in 14 unique cell types (Figure 4D). Seven of these phenotypes are matched between both technologies: B cells, BnT cells, T cells, DCs, macrophages, neutrophils, and epithelial tumor cells.

**Figure 6:**
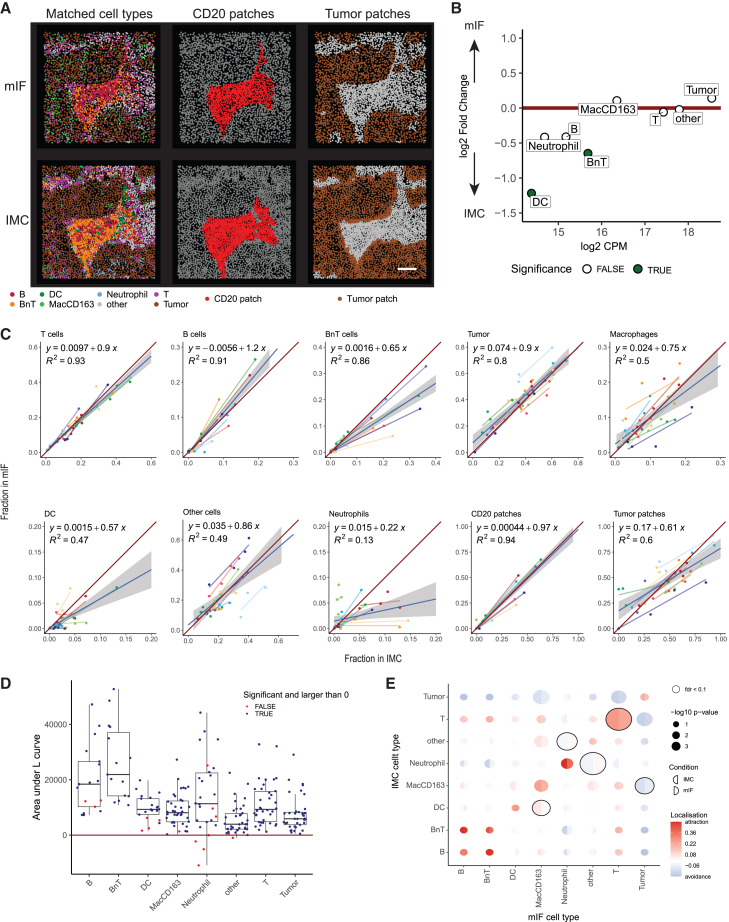
Cell-type and spatial feature comparison between mIF and IMC (A) Matched image from IMC (top) and mIF (bottom) colored by cell phenotype (left), CD20 patches (middle), and tumor-stroma compartment (right). Scale bar: 100 μm. (B) Differential abundance of matched cell types in mIF vs. IMC images (*n* = 38). Results with a false discovery rate (FDR) < 0.05 are colored in green. (C) Scatterplots comparing the fractions of each cell type per image in IMC (*x* axis) against mIF (*y* axis). (D) The area between the calculated and theoretical L functions for the respective cell types in mIF and IMC. Each dot represents one cell type per IMC-mIF image pair. Blue points indicate cells that significantly co-localize in mIF and IMC compared to random. Red points indicate comparisons with negative area or for which a maxium absolute deviation (MAD) test did not result in significant co-localization (*p* > 0.01) (STAR Methods). (E) Results showing the spicyR differential co-localization test. Circles are split into results from IMC (left side of circle) and mIF (right side of circle) indicating whether cells are significantly clustered within the modality. Cell types that show significantly different co-localization between mIF and IMC (FDR < 0.1) are indicated by the black lines around the circles.

To compare the data from mIF and IMC, we aligned the acquired IMC ROIs with the matched regions on the mIF slides using napari (STAR Methods), identified cells in matched regions, and compared their properties (e.g., cell type) between the aligned images (Figure 6A). We observed larger numbers of segmented cells in IMC compared to mIF (Figure S6A), possibly due to differences in resolution and Mesmer detecting more cells than IFQuant (Figure S1B). We observed smaller areas of the segmented cells in IMC compared to mIF (Figure S6B), possibly due to the fact that IFQuant uses Voronoi tessellation for whole-cell detection and tends to merge cells (Figure S1C), and Mesmer tends to detect more cells, thereby possibly underestimating cell size.

We next compared the abundance of cell types between mIF and IMC across all aligned images (STAR Methods). Besides BnT cells and DCs, which were lower in mIF, we observed no systematic differences between mIF and IMC cell-type abundances (Figures 6B and S6C). DCs are the least abundant cell type in the datasets, and reliable detection across consecutive sections is therefore the most challenging. Neighboring B and T cells may be easier to separate in mIF images than in IMC due to higher resolution, resulting in lower counts for BnT cells in mIF.

Linear regression analysis was performed to estimate the variance in cell-type fractions in IMC that can be explained by cell-type fractions in mIF. We observed high similarity in the fractions of T cells, B cells, BnT cells, and tumor cells (R^2^ > 0.8) between mIF and IMC (Figure 6C). Neutrophils showed the lowest similarity between the methods (Figures 6C and S6D), which may be explained by the differences in the markers used for neutrophil detection between IMC (MPO^+^CD15^+^ cells) and mIF (CD15^+^ cells). Using CD15 as a sole marker for neutrophils in mIF hinders the discrimination of spatially overlapping tumor cells and neutrophils from CD15-expressing tumor cells. Overall, we conclude that cells such as lymphocytes and cell types with higher abundance are comparable across mIF and IMC, given the differences in the underlying methods of detection.

B cells, and especially TLSs, are important for immunotherapy prediction^6^^,^^40^^,^^41^ and thus crucial features to be extracted in images. We compared mIF and IMC in terms of the fraction of cells located within B cell patches per image (Figures 6C, S7A, and S7B). We first ensured that B cell patches in the images were found in matched locations using a spatial score based on homogeneous L functions^42^ (STAR Methods). A positive area between the observed and the expected L function indicates that cells reside in the same spatial location on matched IMC and mIF images. In addition, we performed a statistical test to assess if the observed L function is significantly different from the expected L function. Even though the methods of B cell patch detection between mIF and IMC differed, the spatial arrangements of B cells were similar in IMC and mIF images (Figures S7C and S7D). We next assessed whether all matched cell types reside in similar spatial locations on IMC and mIF images (Figure 6D). Overall, for all matched cell types, we observed a positive difference between the observed and expected L functions, indicating that cells show similar spatial distributions between mIF and IMC. For some images, DCs and neutrophils did not show similar spatial distributions, which is in line with the results of the differential abundance test and linear regression analysis. We performed a related spatial analysis, testing for differences in cell-type/cell-type co-localization between IMC and mIF^43^ (Figure 6E).

Most observed cell-type pairs showed similar clustering in mIF and IMC, with slight differences between T cell pairs, tumor cells with macrophages, macrophages with DCs, and neutrophils with other cell types.

In summary, the abundance and spatial distributions of cell types were similar between IMC and mIF for most cell types, showing the robustness of both analysis pipelines to generate data for downstream analysis.

## Discussion

We present semi-automated, high-throughput workflows for multiplexed imaging of FFPE tumor tissues. Developed within IMMUcan,^12^ these workflows are applied with three mIF and two IMC panels on consecutive tissue sections to assess immune infiltration across five cancer types and enable reproducible analysis of thousands of samples for mIF and IMC over 6 years.

Immune cell spatial distribution varies by tumor type and affects disease progression^44^^,^^45^^,^^46^^,^^47^^,^^48^ and treatment response.^9^^,^^11^^,^^40^^,^^49^^,^^50^ While transcriptomic pan-cancer studies exist, large-scale imaging-based studies, such as those generated within IMMUcan, are lacking. Generating reproducible multiplexed imaging data over years presents challenges, including antibody selection, equipment stability and performance, software changes, IT migrations, and staff turnover. Therefore, large, multi-year projects require a robust exploration and evaluation phase followed by consolidation focused on reproducibility and resource needs. Staff changes require strong documentation since downstream pipeline changes are limited due to the risk of generating batch effects or impracticalities to re-generate or re-analyze data.

We used mIF for whole-slide characterization and IMC for high-resolution profiling of selected regions. mIF has been broadly applied in different tumor indications,^51^^,^^52^^,^^53^ but in practice, it is not yet available, nor is it used in clinical pathology.^54^ Existing commercial software for mIF analysis^55^ typically lacks scalability and customization for large studies. To address this, we developed IFQuant, integrated with the LIMS to support sample tracking, staining/scanning QC, semi-automated analysis, and result standardization. Our comparison of IFQuant with other open-source tools revealed that they could potentially be repurposed for tasks such as those required in IMMUcan but do not allow an out-of-the-box integration in the LIMS. However, the latest release of the PhenoImage HT software allows unmixing of images after scanning, enabling the use of external software such as QuPath^31^ or MCMICRO.^32^

IMC studies to date mostly rely on small cohorts or tissue micro-arrays (TMAs),^8^^,^^9^^,^^10^^,^^46^^,^^56^^,^^57^ and tissue sampling critically affects the detection of rare features or tissue-relevant structures.^15^^,^^16^ In IMMUcan, we image 1 mm^2^ per sample via three ROIs of 600 × 600 μm. This approach may miss rare events and introduce biases between IMC and mIF results, but it enables the capture of key structures like tumor-stroma interfaces or TLSs.

To guide ROI selection at scale, options include pathologist-annotated H&E, region-based sampling, or mIF-guided selection.^35^ In IMMUcan, H&E slides were often too distant from the IMC section for accurate alignment (Figure 1C), and the consecutive mIF section from CHUV, utilizing the harsher TSA protocol, more frequently contained tissue loss. We therefore developed an IF/IMC co-staining protocol utilizing fluorescent-labeled primary antibodies for ROI selection directly on IMC slides, reducing hands-on time while retaining informative signals to select regions of tumor-immune mixing. Manual ROI selection remains one of the most critical aspects of our work, and several future options for automating ROI selection exist. These include extracting tile embeddings from consecutive H&E sections using foundation models (e.g., UNI^58^), followed by clustering and proportional sampling, or training deep learning classifiers on expert annotations. Additionally, the latest generation of IMC instruments (released in 2024) can produce low-resolution preview scans (sampling every 25th pixel) within 30 min, allowing marker-based or data-driven ROI selection. These systems also support high-throughput acquisition with a slide loader, a feature first prototyped within IMMUcan and critical for achieving our scale.

Image analysis included image segmentation, single-cell feature extraction, and cell phenotyping. Existing tools at the project’s start were insufficient, and we reused analysis steps from Windhager et al.,^39^ which we developed in parallel to this work, and advocate reusing existing pipelines (such as MCMICRO,^32^ Galaxy-ME,^59^ or SPACEc^60^) as much as possible in the future. Tools like Nimbus^61^ or VirTues^62^ may further reduce manual QC in the future. However, a comparison of current cell-typing tools using our manually labeled data revealed that classic machine learning tools outperformed numerous recently published cell-phenotyping tools. We speculate that the hierarchical nature of the multi-dimensional gated data is particularly suited for tree-based machine learning methods such as XGBoost or random forest.

We identified seven cell types consistently detected across mIF and IMC, showing high spatial concordance despite region-based IMC vs. whole-slide-based mIF results. IMC offered greater resolution for the detection of immune subsets like exhausted T cells or myeloid subtypes. While cell types in mIF were defined based on thresholds, IMC cell types were predicted using a random forest classifier trained on manually labeled cells from 180 images, potentially explaining some of the observed differences. Discrepancies were noted for rare cell types like DCs due to both technical and biological reasons—e.g., CD15^+^ alone in mIF may misclassify neutrophils, while IMC benefits additionally from MPO^+^. Additionally, matching cells across sections remains difficult for rare populations simply due to tissue sectioning.^63^

In summary, we developed scalable, reproducible pipelines for multiplexed tumor imaging, generating consistent single-cell data across technologies. These workflows support IMMUcan’s goal of studying the immune landscapes across cancers and provide resources—including a dataset of 340,000 annotated cells—for the broader research community. The IMMUcan dataset will enable biological investigations, the development of tools for cross-section cell matching^64^ and cross-modality signal prediction,^65^ and the training of foundation models for quality control, cell typing, and prediction tasks.^62^

### Limitations of the study

The most critical limitations of our study are the frequent manual interventions for IMC and mIF. While efficient, IFQuant still requires frequent manual input. Now that annotated ground-truth data are available in large quantities within IMMUcan, a future alternative could be to train deep learning models to detect tissue anomalies such as necrotic regions or tissue folds. Similarly, the implementation for thresholding performed in IFQuant increased efficiency but is time consuming, and one would ideally replace such manual interventions with normalization strategies as presented in Nimbus^61^ or UniFORM.^66^ However, the use of a deep learning model requires a lot of training data, and any model implementation requires thorough validation and likely variable amounts of manual quality control.

The ROI selection for IMC for each patient was a critical component of our workflow and was performed manually due to several constraints. First, criteria for algorithmic ROI selection were not established at project start and remain poorly defined. Our dataset may contribute to defining such criteria in the future. Second, tumor tissues are often adjacent to normal areas that cannot be distinguished solely based on markers like pan-CK, making automated selection error prone. Manual selection enabled visual inspection of each sample, providing an inherent layer of quality control. However, this approach is time consuming, subjective, and potentially biased, despite our use of standardized selection guidelines.

In conclusion, our workflows present semi-automated solutions for large-scale multiplexed image data generation. With the recent explosion of deep learning-based tools, it seems likely that many of the manual steps will be replaced by automated solutions in the future. However, the specific new challenges that arise with more automation remain to be explored.

## Resource availability

### Lead contact

Requests for further information and resources should be directed to and will be fulfilled by the lead contact, Daniel Schulz (daniel.schulz@uzh.ch).

### Materials availability

This study did not generate new unique reagents.

### Data and code availability

• All data have been deposited at Zenodo: mIF and IMC data to reproduce this publication (Zenodo: https://doi.org/10.5281/zenodo.15855866), mIF images and IFQuant parameters for those images (Zenodo: https://zenodo.org/records/12699335), and the manually annotated IMC images (Zenodo: https://doi.org/10.5281/zenodo.12912567).
• The code for cell type classification of IMC data is publicly available (https://github.com/ImmucanWP7/IMMUcan_IMC_panel_1_cell_type_classifier), the code for the *immucan-roi napari* plugin is publicly available (https://github.com/BodenmillerGroup/immucan-roi), the code for the cell-type benchmarking is publicly available (https://github.com/dav3794/IMC-models), the docker image of IFQuant is publicly available (https://github.com/BICC-UNIL-EPFL/IFQuant), and the code to reproduce this manuscript from the data is publicly available (https://github.com/ImmucanWP7/IMMUcanWorkflow2021) and has been deposited at Zenodo (Zenodo: https://doi.org/10.5281/zenodo.16812658).
• Any additional information required to re-analyze the data reported in this work paper is available from the lead contact upon request.

## Acknowledgments

The IMMUcan project has received funding from the 10.13039/501100010767Innovative Medicines Initiative 2 Joint Undertaking under grant agreement no. 821558. This Joint Undertaking receives support from the European Union's Horizon 2020 Research and Innovation Programme and EFPIA (https://IMI.europa.eu). The SPECTA platform is supported by Alliance Healthcare. Alliance Healthcare will become Cencora. We thank Natalie de Souza for critically reading the manuscript. We thank Alex Berlyand for the mechanical design of the slide loader with robotic arm. We thank Nikolai Alexandrov and Olga Loboda for developing a slide loader extension of CyTOF software and its UI for this project.

## Author contributions

D. Schulz, N.E., S.R., S.T., and R.L. designed the study. J.D. and R.L. developed IFQuant with input from S.T. and S.R. S.R., P.D., L.D., and A.B. performed mIF experiments. J.D., S.R., P.D., L.D., and A.B. analyzed the mIF data. A.L., D. Sandkuijl, and N.P. developed the robotic slide loader with input from D. Schulz. M.D., S.D., and D. Schulz performed IMC experiments. N.E., D. Schulz, and B.P.F. performed IMC data analysis. N.E., J.D., and D. Schulz performed the mIF and IMC comparison. M. Możejko, D.U., and E.S. performed the cell-typing benchmark. J.W. developed the immucan-roi napari plugin. N.G., G.C., and B.B. gave input during project progression. N.E. and D. Schulz wrote the manuscript with input from all authors.

## Declaration of interests

The authors declare no competing interests.

## STAR★Methods

### Key resources table

**Table:** 

REAGENT or RESOURCE	SOURCE	IDENTIFIER
**Antibodies**		

CD15 – mIF	Biolegend	Cat#301902; RRID:AB_314194
panCK - mIF	DAKO	Cat#M3515; RRID:AB_2132885
CD3 - mIF	DAKO	Cat#A0452; RRID:AB_2335677
CD11c – mIF	Cell Marque	Cat#111M-15
CD20 – mIF	DAKO	Cat#M0755; RRID:AB_2282030
CD163 – mIF	Histoline	Cat#MB460
MPO – IMC	Dako	Cat#A0398; RRID:AB_2335676
Histone H3 – IMC	CST	Cat#4499BF; RRID:AB_10544537
SMA – IMC	Thermo Fisher	Cat#14-9760-82; RRID:AB_2572996
CD16 – IMC	Abcam	Cat#ab215977; RRID:AB_2877105
CD38 – IMC	Abcam	Cat#ab176886; RRID:AB_2864383
HLA-DR – IMC	Abcam	Cat#ab20181; RRID:AB_445401
CD27 – IMC	R&D Systems	Cat#AF382; RRID:AB_355338
CD15 – IMC	BioLegend	Cat#301902; RRID:AB_314194
CD45RA – IMC	BioLegend	Cat#304102; RRID:AB_314406
CD163 – IMC	Thermo Fisher	Cat#MA1-82342; AB_2275716
B2M – IMC	CST	Cat#47847
CD20 – IMC	Thermo Fisher	Cat#14-0202-82; RRID:AB_10734340
CD68 - IMC	Thermo Fisher	Cat#14-0688-82; RRID:AB_11151139
IDO1 – IMC	Abcam	Cat#ab245737; RRID:AB_2894840
LAG-3 – IMC	CST	Cat#25848; RRID:AB_2943248
CD3 –IMC	Dako	Cat#A0452; RRID:AB_2335677
CD11c – IMC	CST	Cat#45581BF; RRID:AB_2799286
PD1 – IMC	CST	Cat#86163; RRID:AB_2728833
CD140b – IMC	Abcam	Cat#ab215978; RRID:AB_2894841
CD7 – IMC	Abcam	Cat#ab230834; RRID:AB_2889384
GRZB – IMC	CST	Cat#79903; RRID:AB_3665532
PD-L1 – IMC	Abcam	Cat#ab226766; RRID:AB_3073663
TCF7 – IMC	CST	Cat#85942;RRID:AB_3675764
CD45RO – IMC	BioLegend	Cat#304202; RRID:AB_314418
FOXP3 – IMC	Thermo Fisher	Cat#14-4777-82; RRID:AB_467556
CD278 – IMC	CST	Cat#39740; RRID:AB_3676096
CD8 – IMC	Thermo Fisher	Cat#14-0085-82; RRID:AB_11150240
CAIX – IMC	R&D Systems	Cat#AF2188; RRID:AB_416562
CD33 - IMC	R&D Systems	Cat#MAB11371; RRID:AB_2889385
Ki67 – IMC	BD Biosciences	Cat#556003; RRID:AB_396287
VISTA – IMC	CST	Cat#82119; RRID:AB_3683060
CD40 – IMC	Abcam	Cat#ab228818; RRID:AB_2889383
CD4 – IMC	Abcam	Cat#ab181724; RRID:AB_2864377
CD14 – IMC	Abcam	Cat#ab230903; RRID:AB_2889378
E-cadherin/P-cadherin – IMC	Bection Dickinson	Cat#610182; RRID:AB_397581
CD303 - IMC	R&D Systems	Cat#AF1376; RRID:AB_354762
CD206 – IMC	R&D Systems	Cat#MAB25341; RRID:AB_10890782
Cleaved PARP – IMC	Bection Dickinson	Cat#552596; RRID:AB_394437
panCK-Alexa488 – IF-IMC-co-stain	Thermo Fisher	Cat#53-9003-82; RRID:AB_1834350
CD45-DyLight550 – IF-IMC-co-stain	Novus Biologicals	Cat#NBP2-34528R; RRID:AB_3287685
CD163-Alexa647 – IF-IMC-co-stain	Abcam	Cat#Ab218294; RRID:AB_2943126
Discovery OmniMap anti-rabbit HRP	Ventana	Cat#760-4311
Discovery OmniMap anti-mouse HRP	Ventana	Cat#760-4310

**Biological samples**		

Healthy human PBMCs	Interregionale Blutspende SRK	CHUV

**Chemicals, peptides, and recombinant proteins**		

DAPI	Akoya	Cat#FP1490
Iridium intercalator	Standard Biotools	Cat#201192C
DMSO	Sigma Aldrich	Cat#D2650
IFN-ɣ	Roche	Cat#11040596001
Penicillin-Streptomycin	Sigma Aldrich	Cat#P4333
Phytohemagglutinin	Thermo Fisher	Cat#R30852801
Epredia Cytoblock Cell Block Preparation System	Thermo Fisher	Cat#7401150
Hematoxylin in Harris	Biosystems AG	Cat#41-1011-00
Antibody stabilizing solution	Candor Biosciences	Cat#130125
Tween	Sigma Aldrich	Cat#P9416
Ficoll-Paque-Plus	Fisher Scientific	Cat#11768538
BSA	Sigma Aldrich	Cat#A3059

**Critical commercial assays**		

Cytoblock kit	Thermo Fisher	Cat#7401150
Maxpar labeling kits	Standard Biotools	Cat#201300

**Deposited data**		

mIF and IMC data	This paper	Zenodo: https://doi.org/10.5281/zenodo.13132656
mIF images and IFQuant parameters	This paper	Zenodo: https://zenodo.org/records/12699335
Manually annotated data from IMC	This paper	Zenodo: https://doi.org/10.5281/zenodo.12912567

**Experimental models: Cell lines**		

SW480	CCL-228 TM	ATCC

**Software and algorithms**		

IFQuant	This paper	https://github.com/BICC-UNIL-EPFL/IFQuant
Napari image viewer	Napari consortium^38^	https://napari.org/
Immucan-roi napari plug-in	This paper	https://github.com/BodenmillerGroup/immucan-roi
Steinbock toolkit v0.14.1	Windhager et al.^39^	https://bodenmillergroup.github.io/steinbock/latest/
imcRtools	Windhager et al.^39^	https://www.bioconductor.org/packages/release/bioc/html/imcRtools.html
cytomapper	Eling et al.^67^	https://www.bioconductor.org/packages/release/bioc/html/cytomapper.html
Caret	Kuhn et al.^68^	https://topepo.github.io/caret/
SpicyR	Canete et al.^43^	https://www.bioconductor.org/packages/release/bioc/html/spicyR.html
Spatstat	Badeeley et al.^69^	https://spatstat.org/
edgeR	Robinson et al.^70^	https://www.bioconductor.org/packages/release/bioc/html/edgeR.html
Bluster	Bioconductor	https://www.bioconductor.org/packages/release/bioc/html/bluster.html
*S3segmenter* v1.5.6	–	https://github.com/HMS-IDAC/S3segmenter
Segment Anything for Microscopy v.1.3.1	Archit et al.^25^	(https://github.com/computational-cell-analytics/micro-sam
Cellpose v3.1.1.1	Pachitariu et al.^28^	https://github.com/MouseLand/cellpose
InstanSeg v0.0.8	Goldsborough et al.^30^	https://github.com/instanseg/instanseg
StarDist v0.9.1	Weigert et al.^26^	https://github.com/stardist/stardist
DeepCell v0.12.10	Greenwald et al.^27^	https://github.com/vanvalenlab/deepcell-tf
UnMICSTv2.7.7	Yapp et al.^29^	https://github.com/HMS-IDAC/UnMicst
Astir	Geuenich et al.^22^	https://github.com/camlab-bioml/astir
Celesta	Zhang et al.^20^	https://github.com/plevritis-lab/CELESTA
Starling	Lee et al.^23^	https://github.com/camlab-bioml/starling
Stellar	Brbić et al.^19^	https://github.com/snap-stanford/stellar
Bio-formats command line tools v6.6.	Linkert et al.^71^	https://bio-formats.readthedocs.io
libvips	Cupitt et al.^72^	https://www.libvips.org
EBImage	Pau et all.^73^	https://bioconductor.org/packages/release/bioc/html/EBImage.html
igraph	Csardi et al.^74^	https://igraph.org/
data.table	Barrett et al.^75^	https://r-datatable.com
NMF	Gaujoux et al.^76^	https://CRAN.R-project.org/package=NMF
geometry	Habel et al.^77^	https://CRAN.R-project.org/package=geometry
sp	Pebesma et al.^78^	https://CRAN.R-project.org/package=sp
IIPimage	Pillay, R^79^	https://iipimage.sourceforge.io
OpenSeadragon	–	https://openseadragon.github.io/

**Other**		

Code for cell type benchmark	This paper	https://github.com/dav3794/IMC-models
Code to generate figures from this manuscript:	This paper	https://doi.org/10.5281/zenodo.16812658
Code for cell classification in IMC	This paper	https://github.com/ImmucanWP7/IMMUcan_IMC_panel_1_cell_type_classifier

### Experimental model and study participant details

#### Human subjects

Clinico-pathological data and samples were collected according to the EORTC-SPECTA protocol (NCT02834884). All patients provided written informed consent at the time of sample collection for molecular and cellular analysis. The IMMUcan project was approved by several ethical committees; see below for the main recruiting sites: Commissie voor Medische Ethiek ZNA, Belgium on 10/03/2021; Comite de protection des personnes "Nord-Ouest I", France on 02/12/2019; Ethik-Kommission der Medizinischen Universität Wien, Austria on 15/04/2020; Cyprus National Bioethics Committee (CNBC), Cyprus on 29/06/2023; Comissäo de Ética CHUPorto/ICBAS, Portugal on 03/11/2021; CES do IPO Porto, Portugal on 11/03/2021. Patient sample 10074349/IMMU-CRC1-0410 was a distant metastasis. Patient material in IMMUcan is distributed in the form of FFPE blocks, which are cut into individual 4 μm thick sections (Figure 1C). Individual cuts are mounted on microscopy slides, which are sent to the individual partners in roughly four shipment batches per year. Slides 1, 4 and 5 are stained with three panels for mIF and slides 2 and 3 are stained with two panels for IMC. The cohort of ten patients included in this study consisted of 50% male and female patients with a mean age of 64 years (median = 62, range = 47–83).

#### Human peripheral blood mononuclear cells (PBMCs)

PBMCs were prepared from anonymized blood donations from the Interregionale Blutspende SRK from the hospital CHUV in Lausanne.

#### Cell lines

The human SW480 colon cancer cell line (CCL-228 TM) was purchased from ATCC.

### Method details

#### Generation of positive cytoblock controls

Cytoblocks controls combined PBMCs and established SW480 colon cancer cell line. PBMCs were isolated from patient blood by density gradient using Ficoll-Paque-Plus (Fisher Scientific) and immediately cryopreserved in 90% HS (Human Serum) and 10% DMSO (dimethyl sulfoxide) in liquid nitrogen. SW480 cells were thawed and cultured for 2 weeks in two T75 Flask according to ATCC recommendation to obtain tumor cells in exponential growth phase. IFNg (Roche, #11040596001) was added at 200 ng/mL to one SW40 Flask for 24h to induce PDL1 expression. In parallel, PBMCs were thawed and seeded in RPMI 1640 with 8% filtered human serum plus 1% penicillin/streptomycin at a concentration of 1x10^6^ cells/mL with (activated) or without (non-activated) 500ng/mL of PHA (Thermofisher #R30852801) incubated for 48h at 37°C and 5% CO_2_. SW480 tumor cells were harvested from the two T75 Flasks and mixed with either activated or non-activated PBMC (1:1) to a final concentration of 5x10^7^ cells and centrifuged for 5 min at 400xg. Dried cell pellets were resuspended and fixed with 20X Volume of Shandon Formal Fixx solution (Fisher Scientific #7401150) and prepared using the Cytoblock kit (Thermofisher #7401150) following manufacturer recommendation. Briefly, 10x10^6^ cells were mixed with one drop of reagent 2, one drop of reagent 1 plus two drops of Hematoxylin in Harris (Biosystems AG # 41-1011-00) to get a semi-solid mass. The fixed cell mixture was enclosed in a biopsy bag (Leica # 3801085) and in a universal cassette with foam pads (Biosystems AG # 81-0023-00 & # 81-0241-00) for inclusion using the biopsy program from the Vaccum Infiltration Processor system (Sakura tissue embedding console system). Paraffin blocks of the tissue mixtures were then generated (Tissue-Tek TEC 5) for downstream sectioning.

#### mIF staining procedures

An mIF antibody panel was designed to detect immune cell populations. It contains antibodies against CD15: phenotypic marker of neutrophils, CK: tumor marker, CD163: phenotypic marker of macrophages, CD11c: phenotypic marker of dendritic cells, CD20: phenotypic marker of B cells and CD3: phenotypic marker of T lymphocytes. Antibody references and staining conditions are available in Table S1.

Multiplexed staining was performed on tissue sections of 4 μm thickness on automated Ventana Discovery Ultra staining module (Roche). Slides were placed on the staining module for deparaffinization, epitope retrieval (64 min at 98°C) and endogenous peroxidase quenching (Discovery Inhibitor, 8 min, Ventana). Each round of staining included non-specific site blocking (Discovery Goat IgG and Discovery Inhibitor, Ventana), primary antibody incubation and secondary HRP-labeled antibody incubation for 16 min with Discovery OmniMap anti-rabbit HRP (Ventana, # 760–4311) or anti-mouse HRP (Ventana, #760–4310). Covalent dye labeling was then performed using the OPAL reactive fluorophore detection (Akoya Biosciences, Marlborough, MS, USA) for 12 min followed by subsequent heat denaturation of the antibodies for a next round of staining. The mIF stained slides were scanned using the PhenoImager HT (Akoya) with the MOTiF mode, allowing whole slide multispectral image acquisition. The scanner outputs a multi-channel 8-bit image in QPTIFF format.

#### Antibody conjugation for IF/IMC co-stain

Antibodies for IMC were conjugated following the manufacturer’s protocol using Maxpar labeling kits (Standard BioTools). After conjugation the antibodies were stored at the highest possible concentrations but maximally 500 μg/mL in tris-based stabilizing solution (Candor Biosciences) at 4°C.

For IF/IMC co-staining, Alexa Fluor 488 conjugated antibodies against pan-cytokeratin (Thermo Fisher Scientific # 53-9003-82), DyLight 550 conjugated antibodies against CD45 (Novus Biotechnologicals #NBP2-34528R), and Alexa Fluor 647 conjugated antibodies against CD163 (Abcam #ab218294) were purchased and stored at 4°C.

#### IMC antibody panel generation and storage

A large antibody staining mix sufficient for the staining of roughly 500 slides was prepared. The required amounts of metal-labeled antibodies were mixed and diluted with staining buffer (TBS, pH 7.6, 3% BSA, 0.1% Tween) to a predefined concentration (Table S1). The mix was aliquoted into differently sized aliquots and aliquots stored at −80°C until further use.

#### IMC staining

A detailed protocol can be found here (https://www.protocols.io/view/imaging-mass-cytometry-antibody-staining-5qpvo5w2dl4o/v1). Briefly, FFPE slides were removed from −80°C storage and kept at room temperature for at least 10 min to equilibrate to room temperature, deparaffinized and rehydrated and stored in TBS (pH 7.6). To reduce the required amount of antibody solution slides were gently tapped sideways and immediately circled with a PAP-Pen to provide a hydrophobic barrier. Slides were then treated with blocking buffer (TBS with 3% BSA and 0.1% Tween) for 1 h at room temperature. Antibody staining mix aliquots were removed from −80°C and thawed on ice. Fluorescent-labeled antibodies were added to the mix of metal-labeled antibodies (Table S1**)** and gently mixed. Blocking solution was removed from the slides and roughly 30–80 μL of staining mix was added to each slide depending on the size of the tissue. Slides were incubated at 4°C overnight in a wet chamber. The next day slides were washed in TBS and roughly 100–200 μL of a mix of Iridium intercalator (1 μM) and Hoechst solution (2 μg/mL) were added to each slide. Slides were incubated for 5 min in a wet chamber at room temperature and washed with TBS. Slides were then briefly dipped in ddH_2_O and then dried under air flow.

#### Slide-scanning for IF/IMC co-staining and panorama generation

The dried slides after staining were scanned within 48 h with a Zeiss AxioScan with 10× magnification and filters for DAPI, 488, 555 and 647 using the ZEN software.

Subsequently, brightfield scans of prominent tissue areas (partial Panoramas) were generated of the same slides using the Hyperion+ Imaging System (Standard BioTools) with a robotic arm (Meca500, Mecademic) for automatic slide loading. The system is a prototype, was developed with Standard BioTools (former Fluidigm) and required changes to the hardware and software of the Hyperion+ Imaging System. Briefly, after alignment the robotic arm could insert single microscopy slides from a slide hotel into the ablation chamber and place them back to the hotel. The hotel had a maximum capacity of 40 slides. For batch mode, either panoramas or acquisitions had to be defined for each slide of the hotel and could then be run in batch mode for up to 40 slides consecutively.

#### IMC region selection using the immucan-roi plugin for napari

Panoramas (stored in the MCD files of each slide) and immunofluorescence whole slide scans (stored in CZI format) of each patient sample were used as input for the open source immucan-roi plugin for the napari image viewer.^38^ While alignment approaches for IMC and mIF measurements have been developed previously,^36^ partial panorama and mIF alignment was not automatable and required us to develop the immucan-roi plugin for the napari image viewer^38^ for landmark based alignment. For each matched pair of CZI and MCD file images a set of minimally 4 corresponding landmark points were selected on each image. Of note, the selection of prominent tissue areas for panorama generation increases the speed at which landmark points can be manually identified. Due to fluorescence-based and brightfield-based image acquisition being performed on the same tissue slide, even single cells could easily be visually matched during landmark selection. Once 4 matched landmark points have been identified, an affine transformation model is applied to align the images.

After image alignment the tool was used to select ROIs on the fluorescence images. Colors and saturations were adapted as needed and regions selected based on the following general criteria: 1) 30–70% tumor content. To fulfill this requirement, regions were mostly placed at interfaces between tumor, stroma and immune cells. 2) Exclusion of necrotic regions based on DAPI stain. 3) If samples were resections, then one ROI was placed on the global front of the tumor and the other ones in the core of the tumor. 4) If CD45 dense regions (potentially TLS) were visible in the whole slide scan one additional ROI was placed on one such region inside the tumor area, if possible. All regions were selected to be of 600 × 600 μm size except for the 4th additional ROI or if the amount of tissue material prohibited the placement of multiple ROIs. A CSV file was then automatically exported for all regions of interest in IMC coordinate system space.

#### IMC data acquisition

For each slide, the ROIs to be acquired with the Hyperion+ Imaging System, defined using the immucan-roi tool, were imported into the corresponding MCD files (created during panorama generation) and saved therein. Identical to the batch panorama mode, a batch acquisition mode was incorporated into the CyTOF software which used MCD files and a linking CSV file as input. Using the robotic slide loader, batches of typically 20 slides were acquired for IMMUcan. The ten samples described in this paper were selected from a range of samples acquired over time. Samples were acquired at 400 Hz with 1 μm resolution.

#### mIF data analysis

mIF image analysis is performed with the IFQuant software. The detailed methodology as well as the list of tools and packages integrated in IFQuant are described in Methods S1. Briefly, the following steps are performed:

(1) Unmixing: Multiplexed immunofluorescence images contain 8 channels (one channel per fluorophore spectral band and one channel for the autofluorescence). Due to the overlapping emission spectra of the fluorophores, the signal of each channel is a mix of signals from all fluorophores and from autofluorescence. With the help of a library of single stained images, IFQuant can subtract the background signal as well as the estimated contribution of the other fluorophores to the signal of each channel. This process creates a multiband pyramidal unmixed TIFF file readable by the IIPImage image server, used only for visualization. All subsequent operations are performed on images that are unmixed on the fly.
(2) Nuclei segmentation: Nuclei segmentation is done on the unmixed image channel with DAPI nuclear staining by applying an adaptive thresholding strategy, followed by the watershed algorithm applied on the distance map to label individual nuclei.
(3) Cell segmentation: In the absence of a cell membrane staining, the cell regions are approximated by simultaneously extending each nucleus region by up to 5 μm or until touching a neighboring nucleus region (Voronoi based segmentation^80^). In addition to nucleus and cell region, we also define the cytoplasm region as the set difference of cell region and nucleus region (Figure S8).
(4) Per cell fluorescence quantification: For each cell, several summary statistics of pixel intensities are computed to quantify the signal of each marker in the whole cell, the cytoplasm and the nucleus regions.
(5) Cell type assignment: IFQuant uses a simple thresholding approach to classify each cell as being positive or negative for each marker. The type of summary statistics used to quantify a marker depends on the marker location and distribution within the cell (some markers including CD3 are surrounding the nucleus whereas others including CK have a more diffuse location in the cytoplasm, Figure S9A). For each marker a score, defined as a combination of region (nucleus, cytoplasm or cell) and a summary statistic over this region, is empirically chosen (Figure S9B). A threshold is manually chosen for each marker and each cell is classified as positive or negative for this marker depending on whether the score is above or below the threshold. To help with the choice of threshold, a web application allows visualizing full resolution unmixed images and dynamically highlighting cells positive for the selected marker, as well as to display thresholds on scatterplots of marker scores for all pairs of markers. A phenotype is finally assigned to each cell using our phenotype key (Table S1), which maps each combination of marker positivity status to a phenotype. Of note, cells can, for example, be positive for CD163 and CD3 due to spatial spill-over or segmentation errors. Since the expression of markers like CD3 can typically be found in a narrow area around the nuclei, while myeloid markers such as CD163 are diffuse and can therefore more easily lead to the staining of neighboring cells, we prioritized markers based on their staining. In this specific example we would label CD3^+^CD163^+^ cells as T cells since we prioritize CD3.
(6) Tissue segmentation: Cytokeratin (CK) is used as a tumor cell marker. A first “naive” tissue segmentation is done by assigning all cells with CK score above the CK threshold to tumor tissue type and all other cells to stroma tissue type. In a second step, groups of less than 5 stroma (respectively tumor) cells connected to a group of more than 10 tumor (respectively stroma) cells are reassigned to tumor (respectively stroma) tissue (Figures S9C–S9F).
(7) TLS detection: As a proxy for TLS, we use patches of B cells (CD20^+^CK^−^ cells) with a local B cells density above 2000 cells/mm^2^ and at least 40 cells. Patches are found using the alpha shape^81^ for the set of B cell positions.
(8) Quality control checks: QC is performed at different steps during the mIF analysis. During staining and scanning, autostainer failures or scanning failures are recorded. During analysis, samples with more than 50% of missing or folded tissues are set to FAIL. Values between 0 and 50% generate a warning. Further, regions such as tumor/healthy, fat tissue or necrotic regions are identified by the operator and exported as annotations. Failures are set in case the entire sample appeared necrotic. For individual markers, single marker failure – missing signal – is reported as a warning and missing signal for more than one marker results in a failure.

All steps are packaged inside the IFQuant web application (Figure S10). A CSV file is generated for each image containing a row for each cell with the summary intensity of each marker, the area of the cell, whether the cell is located in tumor, stroma, or TLS, and its X and Y location.

Reading of the QPTIFF format is performed with the Bio-formats command line tools.^71^ Image manipulation is performed with libvips^72^ (https://libvips.github.io/libvips). The image analysis script is running in the R software (https://www.R-project.org) with the help of multiple libraries.^74^^,^^75^^,^^76^^,^^77^^,^^73^^,^^78^ The web tool is developed in PHP (backend) and in JavaScript (Frontend). The backend uses the SLIM framework (https://www.slimframework.com, version 3). Cell quantification data is loaded and indexed in a SQLite3 database for improved performance. The tiled images are served by the IIPImage^79^ image server, using the Internet Imaging Protocol. The frontend is developed with the VueJS (https://vuejs.org/, version 2) and the Bootstrap (https://getbootstrap.com/, version 4) libraries. The JavaScript image viewer is based on the Openseadragon library (https://openseadragon.github.io/, version 4), with the OpenSeadragonFiltering (https://github.com/usnistgov/OpenSeadragonFiltering) and OpenSeadragonScalebar (https://github.com/usnistgov/OpenSeadragonScalebar) plugins. The FabricJS library (http://fabricjs.com/, version 5) is used to draw annotations on the image. The source code of the web-tool as well as the different components are available as a docker image (https://github.com/BICC-UNIL-EPFL/IFQuant).

#### Comparison of nuclear segmentation methods

For each IMC ROI, nuclear segmentation masks were generated using unmixed mIF DAPI images as input to the following tools: (1) Segment Anything for Microscopy^25^ v.1.3.1. (https://github.com/computational-cell-analytics/micro-sam) using automatic segmentation (micro_sam.automatic_segmentation) with vit_l_lm model. (2) cellpose^28^ v3.1.1.1 (https://github.com/MouseLand/cellpose) with built-in cyto3 and nuclei models using automatic diameter estimation. (3) InstanSeg^30^ v0.0.8 (https://github.com/instanseg/instanseg) with fluorescence_nuclei_and_cells model. (4) StarDist^26^ v0.9.1 (https://github.com/stardist/stardist) with 2D_versatile_fluo model. (5) DeepCell^27^ v0.12.10 (https://github.com/vanvalenlab/deepcell-tf) with Mesmer model (membrane channel set to 0). (6) UnMICST^29^ v2.7.7 (https://github.com/HMS-IDAC/UnMicst) with nucleiDAPI1-5 model and S3segmenter v1.5.6 (https://github.com/HMS-IDAC/S3segmenter).

To evaluate the matching between nuclear segmentation masks obtained with two different methods (method 1 and method 2), the overlap coefficient (Szymkiewicz–Simpson coefficient) Overlap(A,B) = |A⋂B|/min(|A|,|B|) was evaluated for all pairs of nuclei found with method 1 and method 2, where A (resp. B) is the set of pixels associated with the nuclei obtained with method 1 (resp. method 2).

Each nuclei pair with an overlap coefficient >0.5 was considered for comparison and assigned to one of the following classes.

(1) "1 - 1": one nucleus obtained with method 1 overlaps with one nucleus obtained with method 2 and conversely.
(2) "1 - many": one nucleus obtained with method 1 overlaps with many nuclei obtained with method 2.
(3) "many - 1": one nucleus obtained with method 2 overlaps with many nuclei obtained with method 1.
(4) "many - many": many nuclei obtained with method 1 overlap with many nuclei obtained with method 2.
(5) "1 - missing": one nucleus obtained with method 1 does not overlap with any nuclei obtained with method 2.
(6) "missing - 1": one nucleus obtained with method 2 does not overlap with any nuclei obtained with method 1.

#### IMC data preprocessing

MCD files from IMC were processed using the steinbock toolkit (v0.14.1).^39^ First, TIFF files were generated and hot pixel filtered. Segmentation was performed using the Mesmer^27^ implementation within steinbock. Histone H3 and Iridium were used for nuclei detection and E-cadherin, CD3, CD8, CD20, CD163, were used as markers for cellular boundaries. Cellular interactions were quantified as cells that touch after a cell boundary expansion of 4 μm. Finally, the mean marker intensities, cellular area, centroid, major and minor axis length and eccentricity were calculated per cell. One CSV file per image containing the mean pixel intensities, one CSV per image containing the cellular information and one CSV per image containing the neighboring cell information were exported.

#### IMC cytoblock stability analysis

Single cell data was obtained from pre-processing and was analyzed in R. Spillover correction was performed as previously described using the imcRtools and CATALYST packages.^82^^,^^83^ Graph-based clustering using Phenograph^84^ was performed to identify cell types using 45 nearest neighbors (k = 45) and clusters were annotated manually. The fractions of cell types measured in cytoblocks with and without activation were calculated per batch and the coefficients of variation for cell type fractions were calculated across batches (*n* = 11). To compare the marker expression variation across batches, for each cell type we only considered markers expressed in a given cell type to avoid calculating variation of noise (Table S1). We calculated the mean expression of relevant markers for each cell type (*n* = 12) within each batch (*n* = 11). We then calculated the coefficient of variation for each marker per cell type across batches. The final coefficient of variation (CV) for each marker was derived by averaging the CV over cell types. Of note, we calculated CVs first per cell type because the expression of markers across cell types can vary significantly.

#### IMC cell type classification

A total of 179 images from 179 patients spanning the five tumor indications were selected for cell type annotation. The R Bioconductor package cytomapper^67^ was used to label cells via multi-dimensional gating and to inspect gates on images. A detailed sketch of the gating strategy can be found in Methods S2. Slightly over 2000 gates were stored for 14 cell types and a total of 343′236 cells.

All gates were then concatenated and cells that had been labeled multiple times were excluded if they were not of type tumor. Cells that were labeled as tumor and another cell type were finally labeled as the other cell type to preserve immune cells within the tumor compartment which may be tumor marker positive due to spatial marker spillover. A random forest classifier was trained using the caret^68^ package with the cancer indication as a covariate. Briefly, the data was split into training (80%) and test dataset (20%) and a 5-fold cross validation was used to train the model while tuning the mtry parameter. The model was then tested on the test dataset (Figure S4). The pre-trained random forest classifier was applied to all samples stained with panel 1 in IMMUcan and the 10 samples presented here. Cells that show a maximum classification probability for any class below 30% obtained the label “unknown”. The labeled data used for training of the classifier can be found here: https://zenodo.org/records/12912567.

#### Cell typing benchmark

The manually labeled data was split into training (80%) and test (20%) data using the same split as in the original classifier. We trained the following traditional machine and deep learning models for cell typing: Astir,^22^ Celesta,^20^ Starling,^23^ Stellar,^19^ Logistic Regression, Random Forest, XGBoost and Multilayer Perceptron (MLP).

For the Astir, Celesta, Stellar and Starling models, we used the default hyperparameters introduced by the authors. For Starling, a model designed to cluster cells taking into account segmentation errors, known cell type labels were used as the initial assignment of observations to clusters for model initialization. For the general machine learning models (Logistic Regression, Random Forest, XGBoost and MLP), we used the default hyperparameters implemented in the corresponding Scikit-learn and XGBoost library classes. In addition, the tree-based models (Random Forest and XGBoost) were trained with 1000 estimators.

Due to technical difficulties, we did not include CellSighter in our comparison.

The performance of the trained models was evaluated using accuracy, macro F1 metrics, true positive rate (TPR) and false positive rate (FPR) on the test data.

#### IMC sub-clustering

Sub-clusters were generated for B, BnT, CD4 T, CD8 T cells, macrophages and DCs. Each cell type was clustered individually using markers with either known expression on the cell type (e.g., PD1 on T cells) or markers that arise from spatial spillover (e.g., CD20 on DCs in dense B cell areas). The complete list of which marker was used for which cell type is shown in Table S1. The R package bluster was used to cluster cells using shared nearest neighbor graphs with rank based edge weighting and louvain-based community detection. We used 80, 40, 50, 40, 40 and 50 neighbors to cluster B, CD4, BnT, CD8, DC and MacCD163 cells, respectively. Based on marker expression, names were assigned to each cluster leading to potential cluster merging of similar clusters. E.g. B cell sub-clustering revealed 6 clusters of which three had no expression of either Ki67, CD40 or CD27, our B cell relevant markers. Those three sub-clusters were named “B” since they could not be distinguished further. Similarly, sub-clustering B cells and BnT cells revealed clusters that were high for CD40 and had medium expression of CD27 and both those clusters were named “B_act”.

#### IMC data analysis and comparison with mIF

IMC data analysis was performed using R. The imcRtools^39^ package was used to read pre-processed data from steinbock following our workflow.^39^ CD20 patches were calculated based on the neighborhood graph calculated with steinbock and patches were called when consisting of minimally 25 B or BnT cells. Detected patches were expanded by 10 μm to include closely neighboring cells. Tumor patches were also calculated based on the neighborhood graph from steinbock and had to initially consist of 25 tumor cells. We also consider all cells within 25 μm from a tumor patch cell to belong to tumor patches. For comparison with the mIF data, the ROIs from IMC were manually identified in the mIF images using napari. mIF and IMC images were then aligned using a similarity transform with the landmark points obtained from napari. Of note, one image was removed due to imprecise alignment. Matched cell types (*n* = 8) between both datasets were used for direct comparison. Differential abundances of cell type counts between mIF and IMC images were calculated using the edgeR^70^ Bioconductor package (v.4.2.0). Absolute cell type counts were modeled with quasi-likelihood negative binomial (NB) generalized log-linear models. Negative binomial dispersion was estimated using estimateDisp(trend = “none”), and quasi-likelihood dispersion was estimated using glmGLFit(abundance.trend = FALSE; robust = TRUE, legacy = TRUE). Differential abundance of cell types across images (*n* = 38) was then tested with glmQLFTest(), which applies a quasi-likelihood F-test to the fitted model coefficients and results were deemed significant with an FDR <0.05. Spatial associations based on the X and Y coordinates between matched cell types in mIF and IMC images (*n* = 38) from the consecutive sections were calculated using the Lcross function from the sf and spatstat^69^ R packages. We performed a maximum absolute deviation (MAD) test using the mad.test function to assess if the estimated L function wanders outside an envelope around the expected L function generated by calculating the L function of 99 simulated realizations of complete spatial randomness. We consider cell type pairs per image set to be similarly co-localized between mIF and IMC if the area between the estimated and expected L function is larger than 0, and if the MAD test *p*-value is smaller than or equal to 0.01. We used the spicyR^43^ Bioconductor package (v.1.16.4) spicy() function to detect differential cell type/cell type co-localization between IMC and mIF at a false discovery rate threshold of 10%. For the mixed-effects linear model we used images as random effect and modality (mIF and IMC) as fixed effect and tested associations using 20, 50, 100 and 150 μm radii with square windows. Spatial comparisons were performed for single cells for a total of 8′657 B cells, 12′823 BnT cells, 4′369 DCs, 16′086 macrophages, 5′088 neutrophils, 41′858 other cells, 37′284 T cells and 69′432 tumor cells.

### Quantification and statistical analysis

Statistical tests were performed in R version 4.4.0. Details of used statistical tests are given within the method details sections.

## Supplemental information

Document S1. Figures S1–S10 and Methods S1 and S2

Table S1. Antibody panels, phenotype key, and cell-type-relevant markers, related to Figures 2 and 3 and STAR Methods

Document S2. Article plus supplemental information
