# Supplementary material for: Multi-modal image analysis for large-scale cancer tissue studies within IMMUcan
Source: Cell Rep Methods. 2025 Sep 9;5(9):101170. doi: 10.1016/j.crmeth.2025.101170 (PMC12539258; doi:10.1016/j.crmeth.2025.101170)
Supplement: Document S1. Figures S1–S10 and Methods S1 and S2 [file mmc1.pdf]

Cell Reports Methods, Volume *5*

Supplemental information

Multi-modal image analysis for large-scale cancer tissue studies within IMMUcan

Nils Eling, Julien Dorier, Sylvie Rusakiewicz, Robin Liechti, Preethi Devanand, Michelle Daniel, Jonas Windhager, Bruno Palau Fernandez, Sophie Déglise, Lucie Despland, Abdelkader Benyagoub, Marcin Możejko, Dawid Uchal, Ewa Szczurek, Alexander Loboda, Daaf Sandkuijl, Nikesh Parsotam, Henoch S. Hong, Marie Morfouace, Nicolas Guex, George Coukos, Bernd Bodenmiller, Stephanie Tissot, and Daniel Schulz

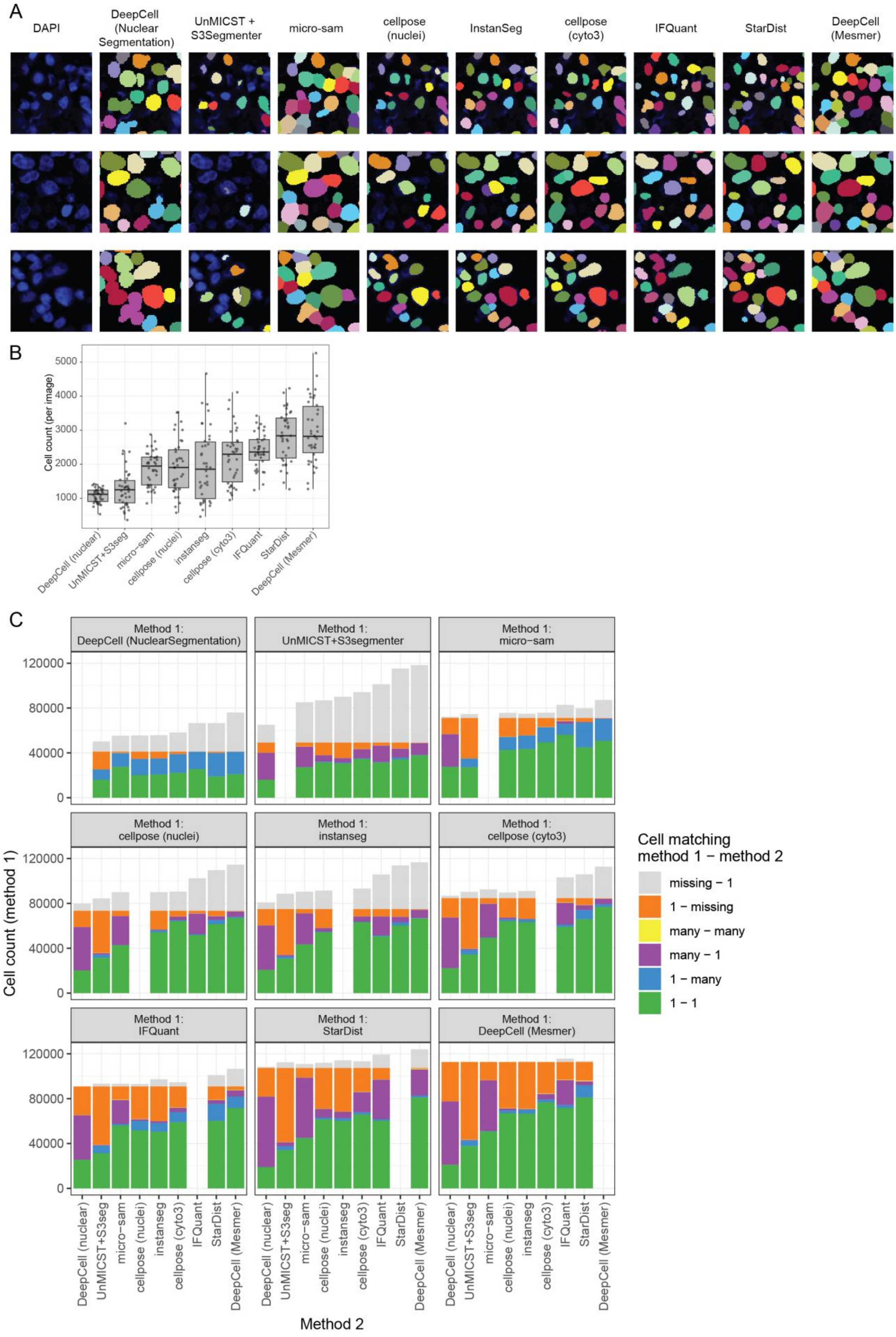
A
DAPI
DeepCell (Nuclear Segmentation)
UnMICST + S3Segmenter
micro-sam
cellpose (nuclei)
InstanSeg
cellpose (cyto3)
IFQuant
StarDist
DeepCell (Mesmer)
B
Cell count (per image)
DeepCell (nuclear)
UnMICST+S3seg
micro-sam
cellpose (nuclei)
instanseg
cellpose (cyto3)
IFQuant
StarDist
DeepCell (Mesmer)
C
Method 1: DeepCell (NuclearSegmentation)
Method 1: UnMICST+S3segmenter
Method 1: micro-sam
Method 1: cellpose (nuclei)
Method 1: instanseg
Method 1: cellpose (cyto3)
Method 1: IFQuant
Method 1: StarDist
Method 1: DeepCell (Mesmer)
Cell count (method 1)
Method 2
Cell matching method 1 − method 2
missing − 1
1 − missing
many − many
many − 1
1 − many
1 − 1

Figure S1: Segmentation algorithm investigation, related to Figure 2.

(A) The DAPI staining is shown on three exemplary image crops per row. The resulting detection of nuclei with individual segmentation tools is shown in columns. **(B)** Distribution of number of nuclei (y-axis) from the matched ROIs (points) obtained with each method (x-axis) applied on unmixed mIF DAPI images. **(C)** Stacked barplots showing the number of nuclei (y-axis) obtained with method 1 (panel) colored by the type of matching (color) with nuclei obtained with method 2 (x-axis) **(Methods)**.

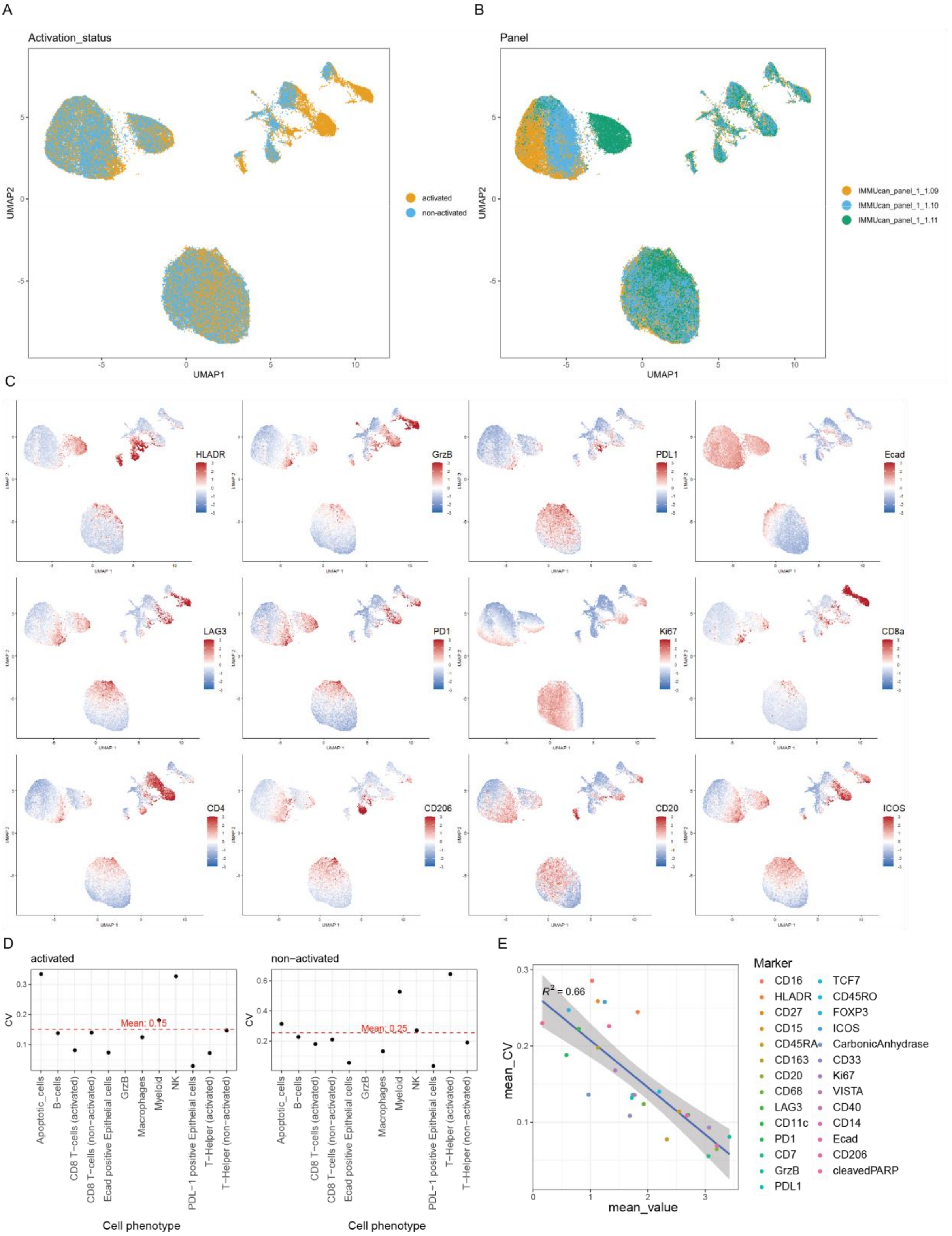

Figure S2: Cytoblock based IMC measurement stability, related to Figure 3.

(A) UMAP of single cells from cytoblocks colored by the activation of the PBMCs in the cytoblocks. **(B)** UMAP as in A colored by the antibody panel mix used for staining. **(C)** UMAP of single cells from cytoblocks colored by the z-scaled expression of markers. **(D)** For each cell phenotype detected in the cytoblocks the mean coefficient of variation (CV) of the detection frequency over all time points (sample batches) is shown (black points) for activated cytoblocks on the left and for non-activated cytoblocks on the right. The Horizontal red dashed lines indicate the mean observed CV for all cell phenotypes. **(E)** scatterplot of the observed mean expression of each marker on the x-axis and the average coefficient

of varation for each marker calculated across batches on the y-axis. Individual points are colored by the respective marker.

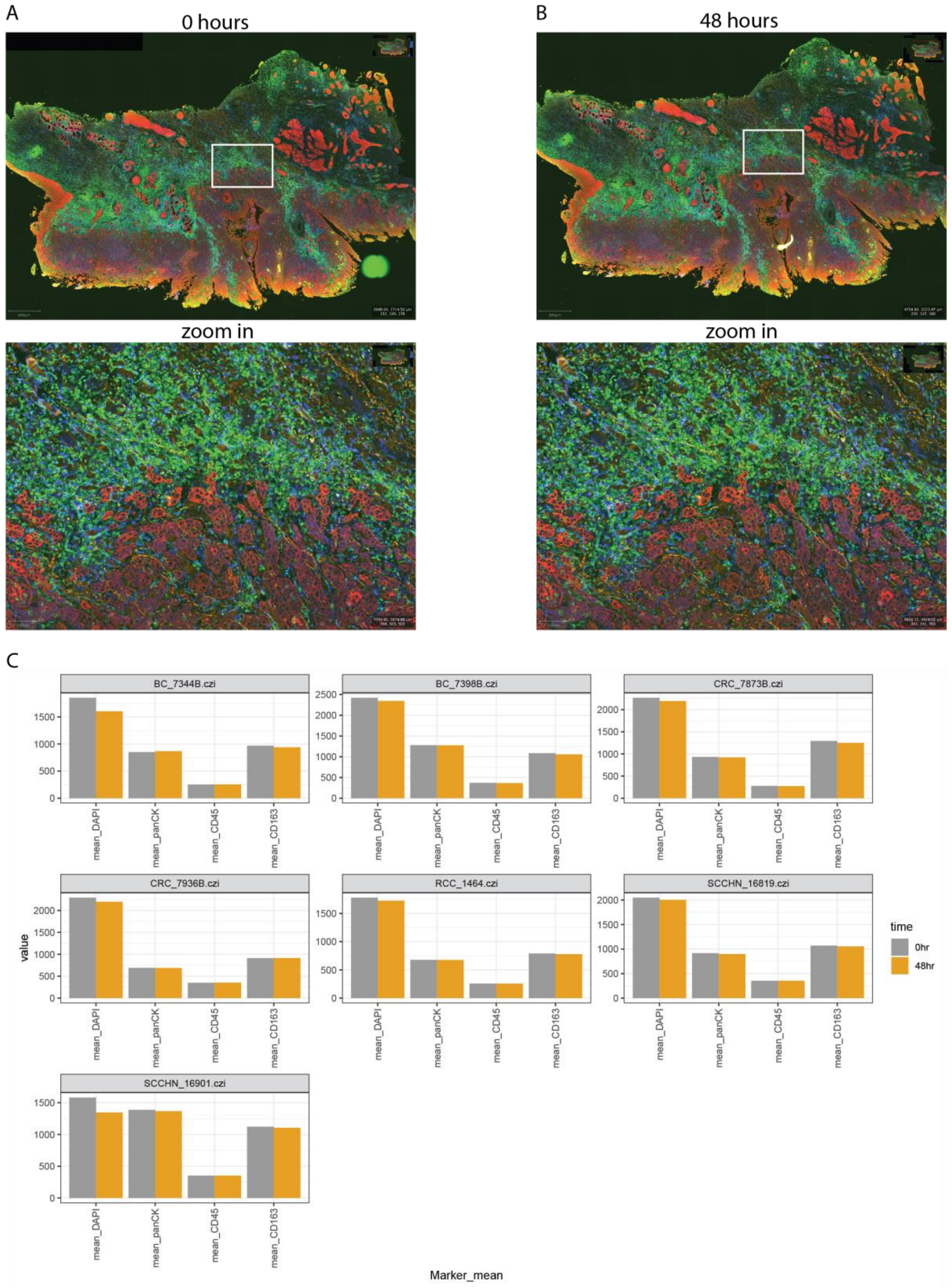

Figure S3: Fluorescence signal stability in dry format, related to Figure 4.

Three color fluorescence images recorded immediately after drying (**A**) and 48 hours after drying (**B**). Zoom ins are shown on the bottom. Scale bars on the top row are 800 µm and on the bottom row 100 µm. **C.** The fluorescence intensity in segmented single cells for 7 individual tumors at 0 and 48 hours after drying for Dapi, pan Cytokeratin, CD45 and CD163 is shown.

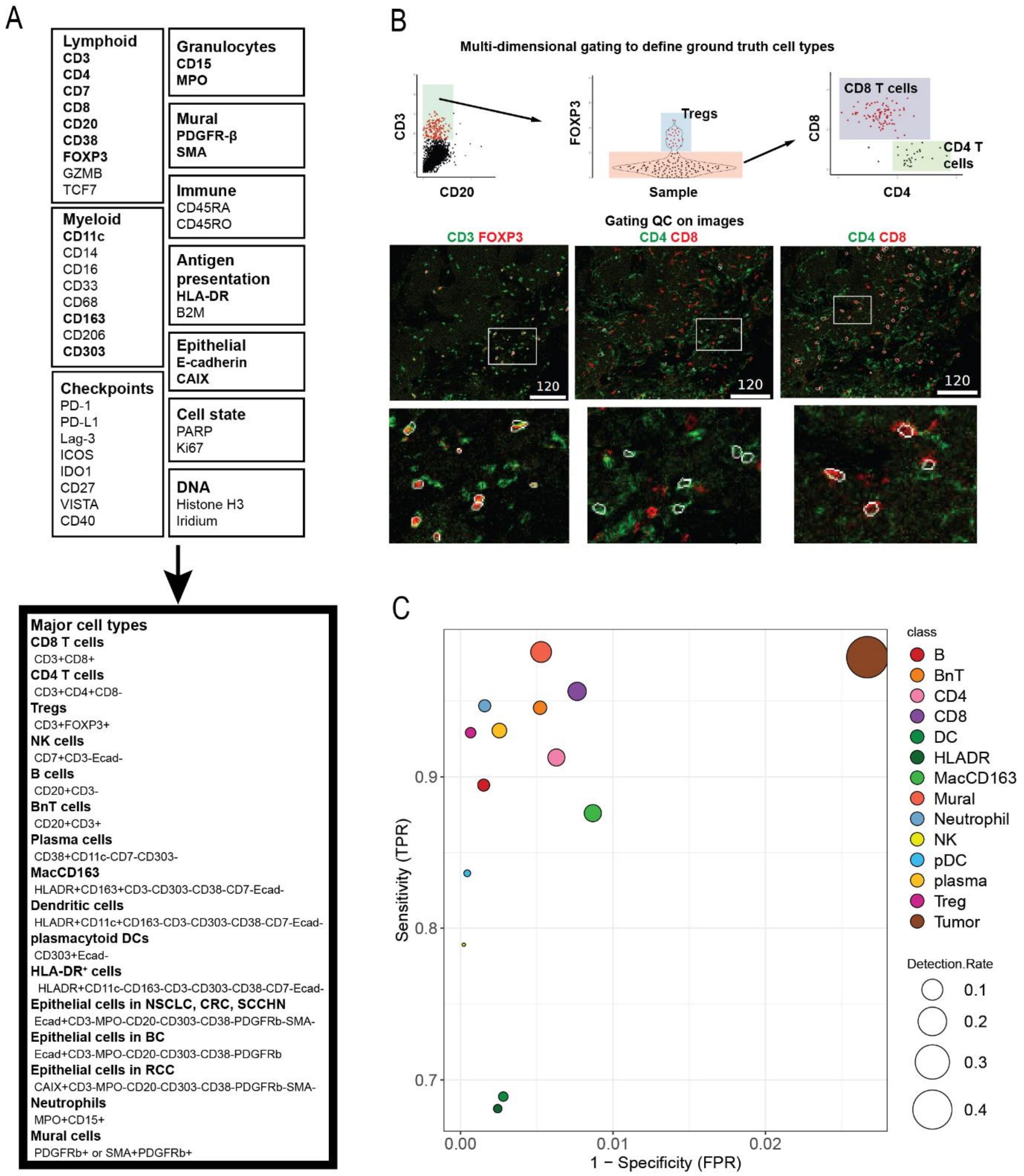

Figure S4: Random forest classifier for cell types, related to Figure 4.

A. Markers in IMC panel 1 are functionally grouped and the major cell types listed. **B.** An exemplary gating scheme is shown on top for regulatory, CD4 and CD8 T cells. Arrows depict the population of cells that moves to the next gate. Named gates represent the final population. **C.** True positive rates (TPR) and false positive rates (FPR) for the detection of individual cell types are shown based on a hold out test data set.

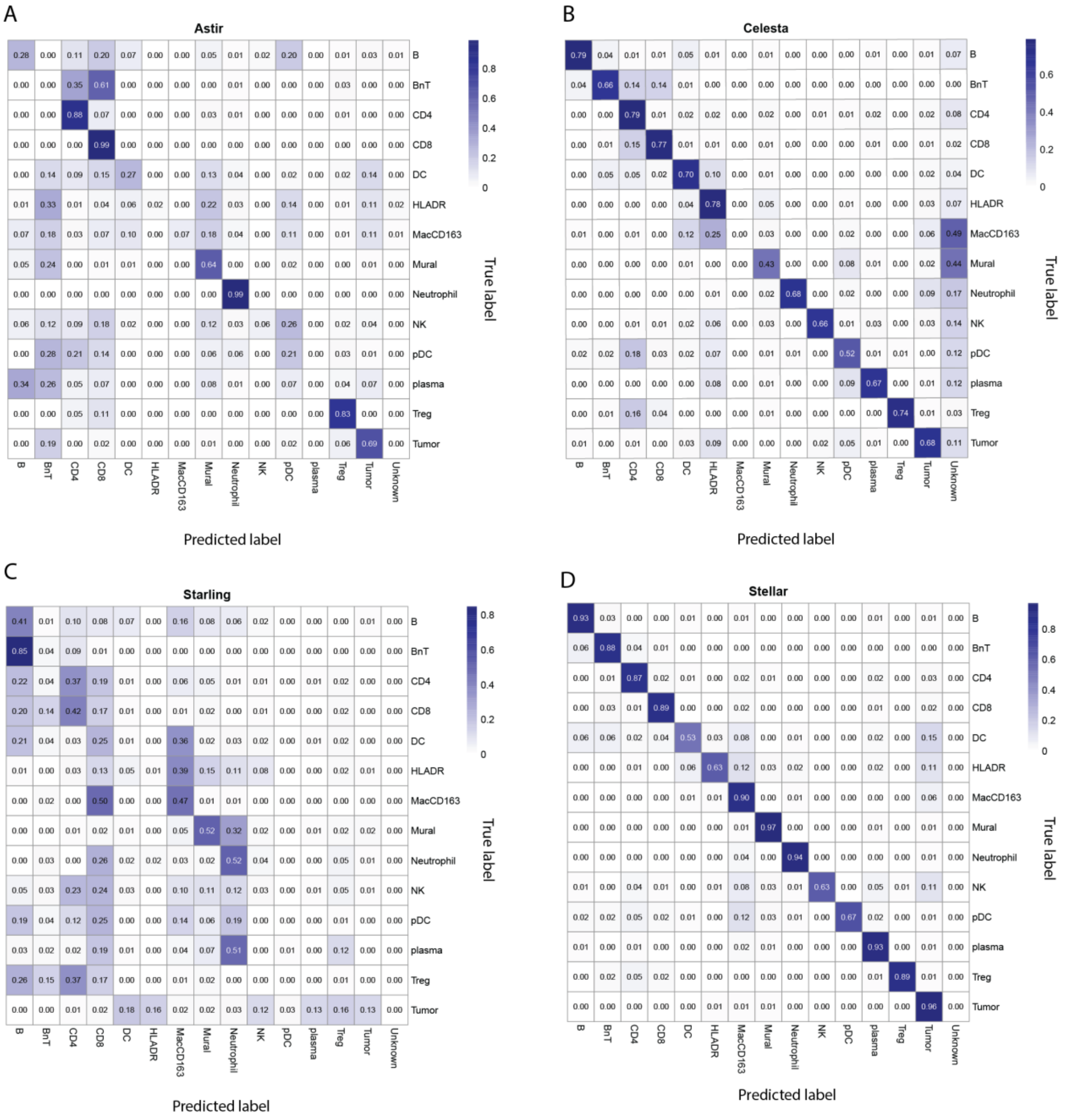

Figure S5: Confusion matrices for different cell typing tools, related to Figure 5.

Confusion matrices with the true cell type labels in rows and the predicted labels in columns for Astir (**A**), Celesta (**B**), Starling (**C**) and Stellar (**D**). Each cell indicates the proportion of true label falling into the class of the predicted label.

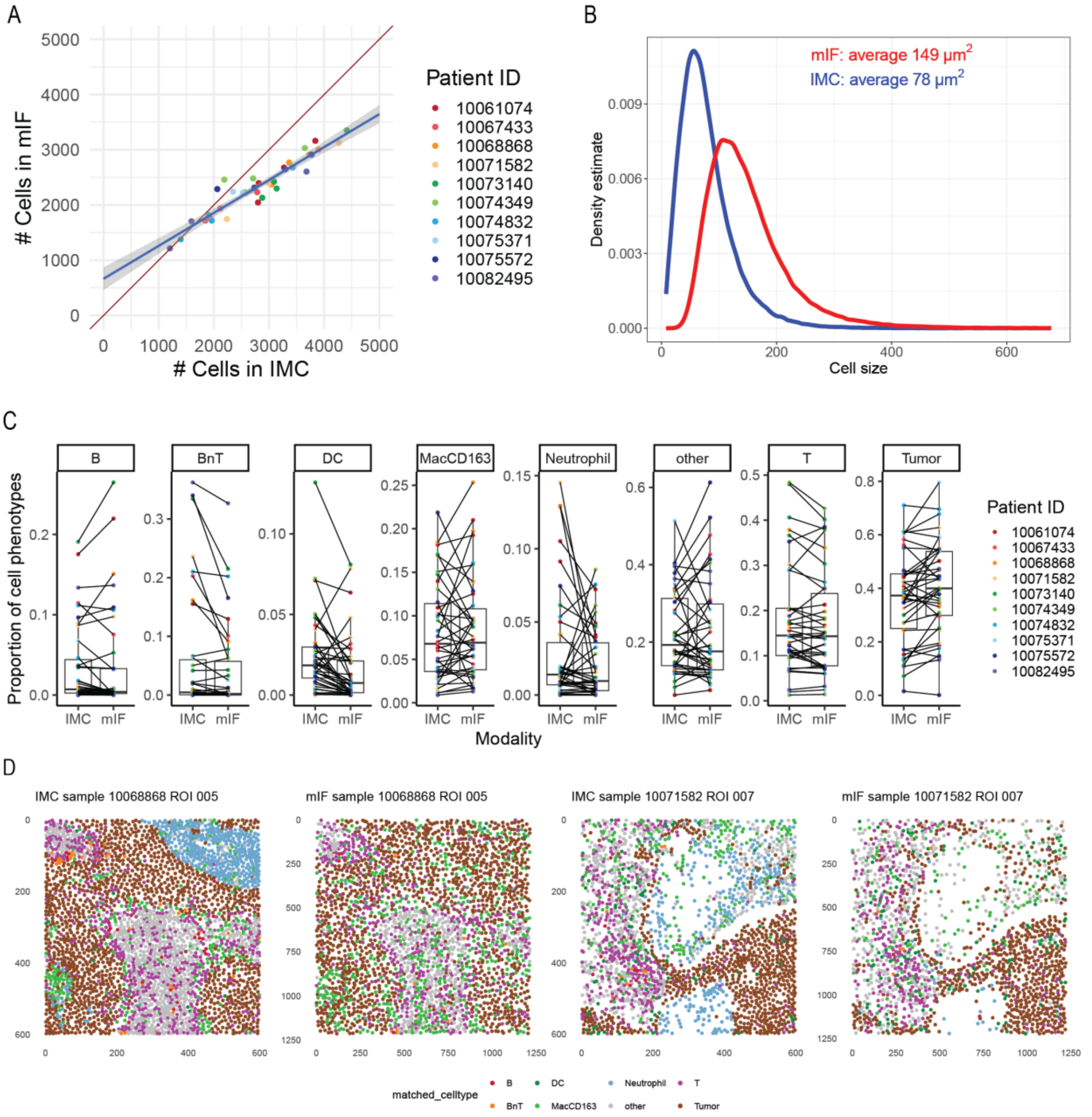

Figure S6: Comparison of IF and IMC, related to Figure 6.

(A) Scatterplot showing the number of segmented cells in IMC on the x-axis and in mIF of the matched region on the y-axis. Images are colored by patient ID. **(B)** Distribution of the single cell area in mIF (red) and IMC (blue). **(C)** For each matched cell phenotype the proportion of the phenotype among all cell phenotypes is compared between mIF and IMC for matched images. **(D)** Examples of single cells on images to highlight differences in neutrophil detection. The first and third images are from IMC and the second and fourth from the matched ROIs in mIF. Each cell is represented by a point reflecting the center of the cell and colored by the respective cell phenotype. Dimensions of the images are given on the x and y axis.

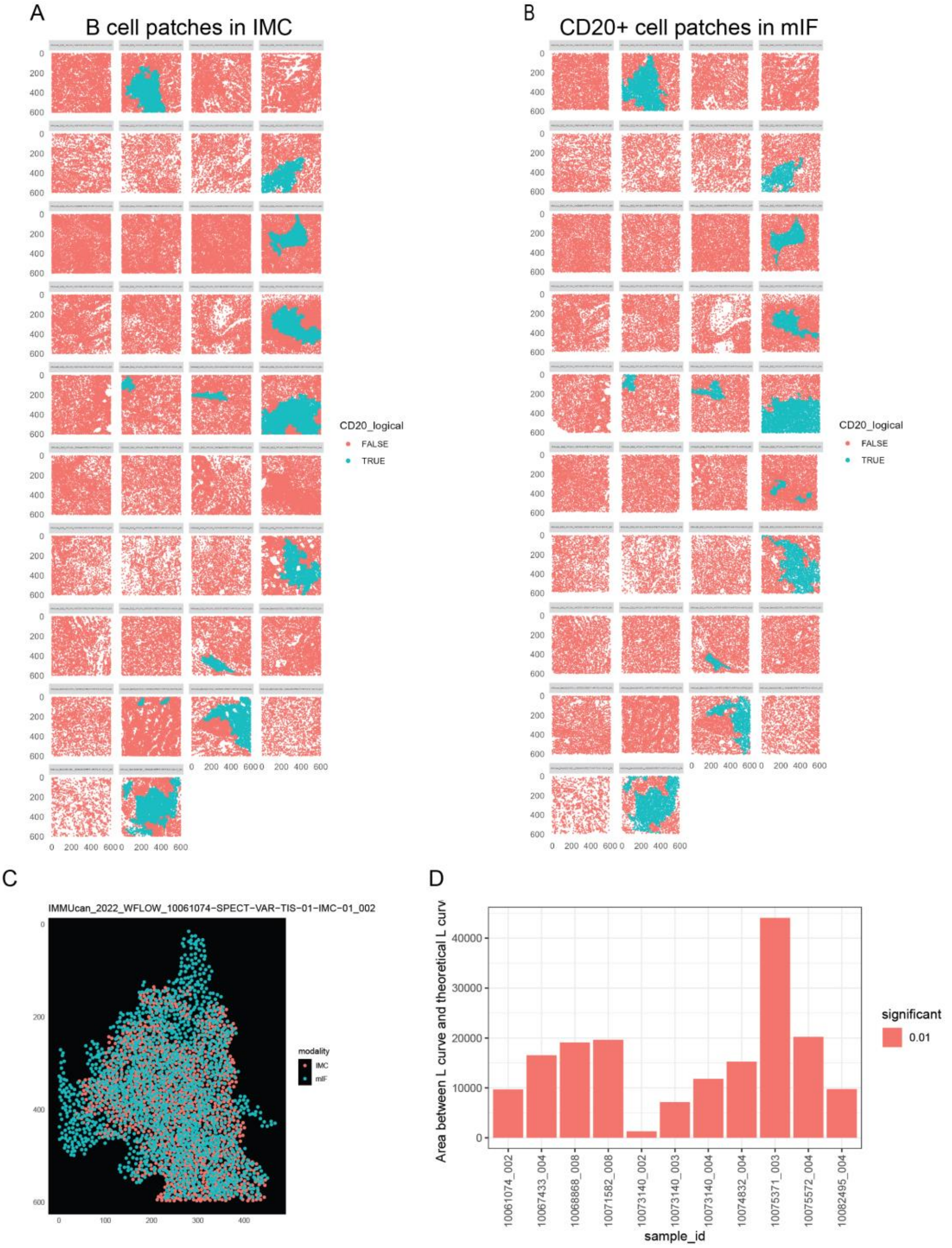

FigureS7: spatial comparison of mIF and IMC, related to Figure 6.

(A) B cell patch detection in IMC based on CD20 positive cells. Cells are depicted as points by the center **(B)** B cell patch detection in mIF based on CD20 positive cells. **(C)** Center points from B cells of one matched image from mIF (green points) and IMC (red points) were overlayed to highlight the matched location of the cells in the image. The image corresponds to the image from the top row, second column in A and B. **(D)** Results from Lcross functions applied to B cells for mIF and IMC are shown as the area between the calculated and the theoretical L curve. Positive values indicate that cells are more clustered as expected if cells were randomly distributed in the images. Significances were calculated based on 100 envelope simulations.

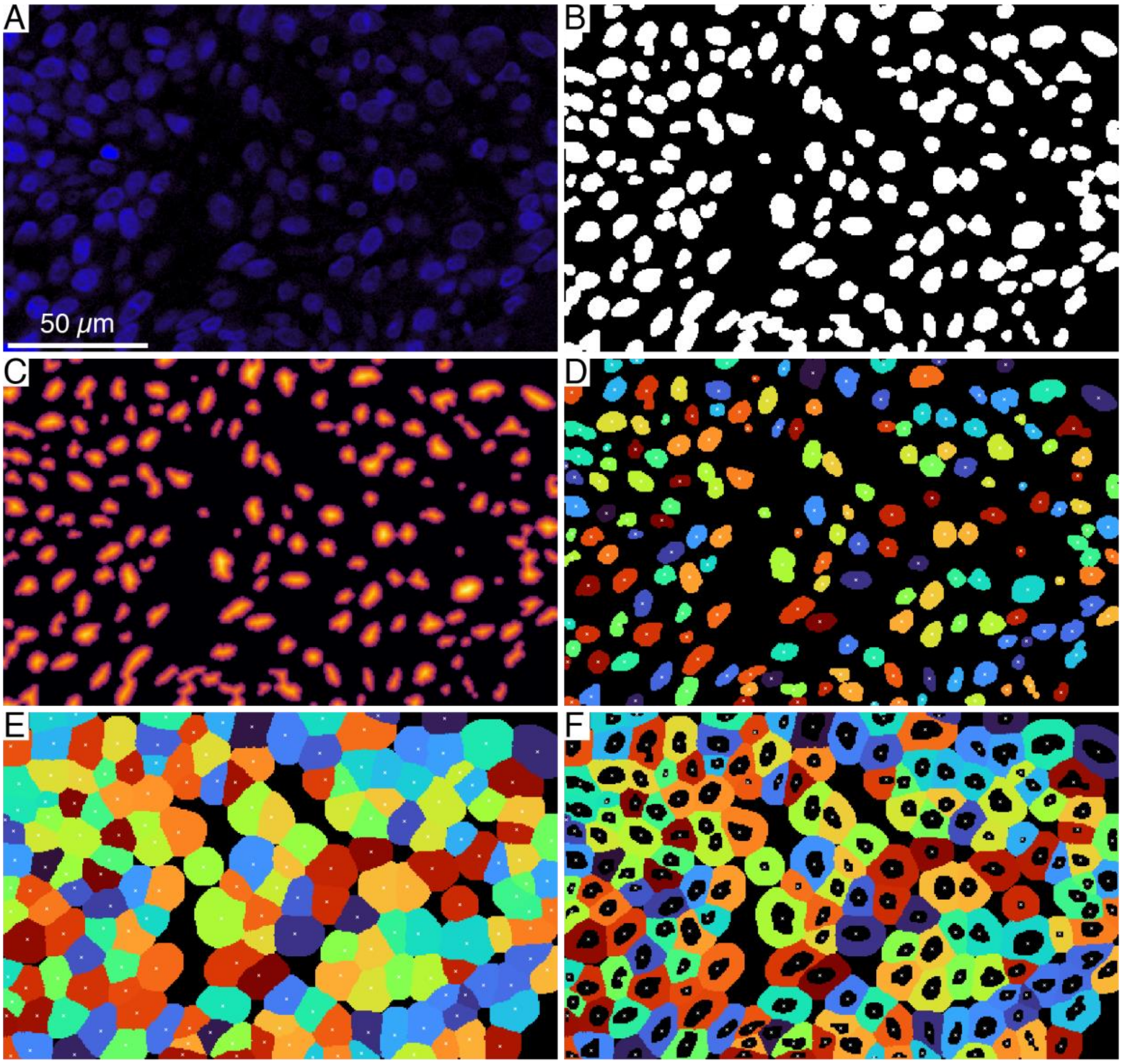

Figure S8: Nuclei & cell segmentation, related to STAR Methods.

(A) DAPI channel. **(B)** Nuclear mask obtained by adaptive thresholding. This mask contains value 1 (white) for nucleus regions and 0 (black) for background. **(C)** Distance map of the nuclear mask colored from black (distance 0) to yellow (maximum distance). **(D)** Nuclear mask after cleaning (colored by cell ID, background in black) with nuclei centers (white crosses). **(E)** Cell regions (colored by cell ID, background in black) approximated by Voronoi based segmentation with nuclei centers (white crosses). **(F)** Cytoplasm regions (colored by cell ID, background in black) with nuclei centers (white crosses).

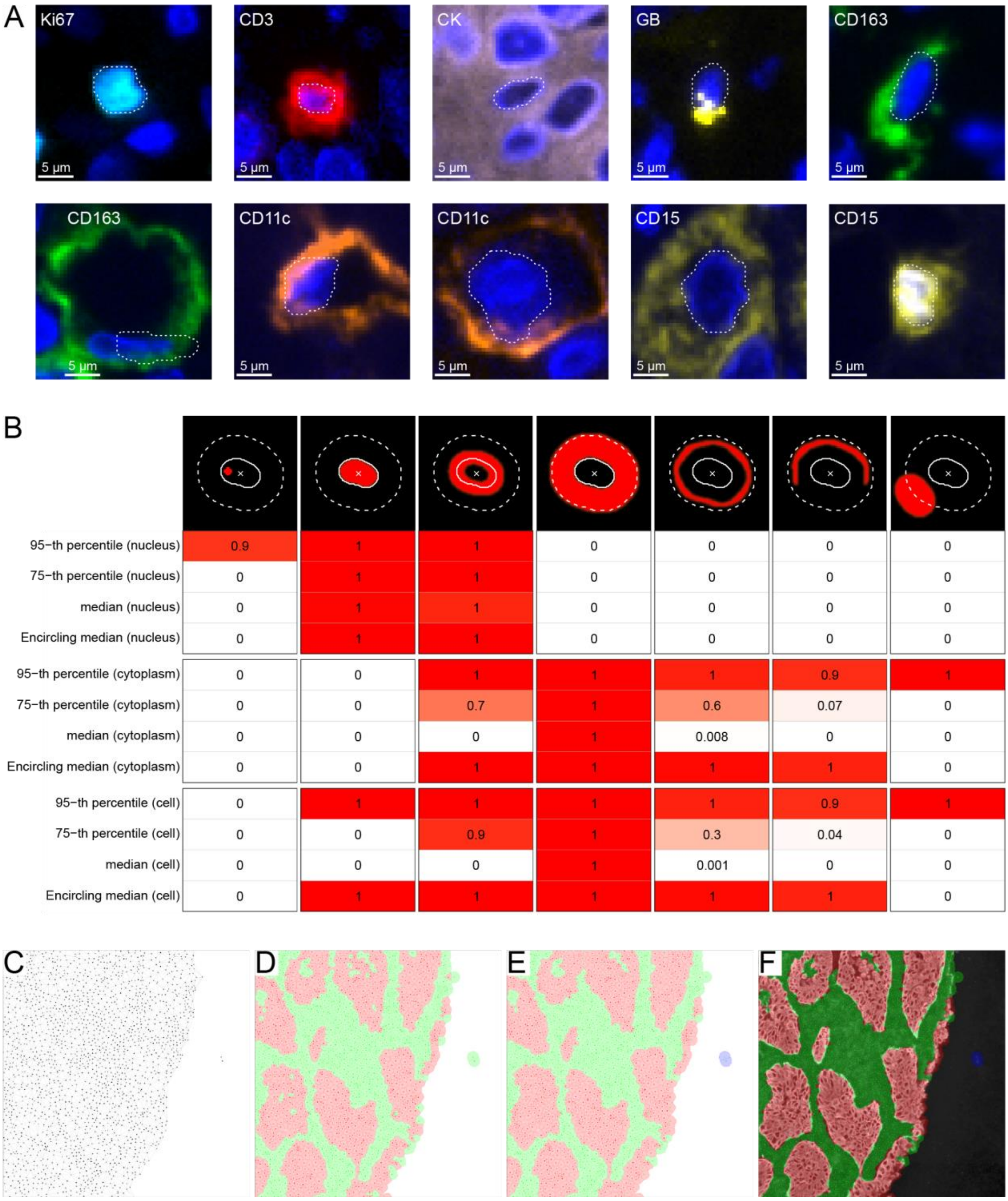

Figure S9: Spatial distribution of fluorescence, single cell quantifications and tissue segmentation, related to STAR Methods.

(A) Spatial distribution of fluorescence for various markers. Each panel shows DAPI channel (blue) with nucleus border (dashed white) together with the fluorescence for specific marker (label in upper-left corner): Ki67 (cyan), CD3 (red), CK (pink), GB (yellow), CD163 (green), CD11c (orange) and CD15 (yellow). **(B)** Score obtained with various combinations of summary statistics and regions (rows) evaluated on archetypal spatial distributions of fluorescence (columns). Images on top show distributions of fluorescence, colored from black (fluorescence=0) to red (maximum fluorescence=1), with nucleus center (white cross), nuclear region boundary (plain lines) and cell region boundary (dashed line). **(C)** Delaunay triangulation. Edges with length above 40 µm are not shown. Vertices (black dots) correspond to nuclei centers. **(D)** Voronoi tessellation after clipping each cell to a maximum

distance to nucleus center of 15 μm. Nucleus centers (dots) and Voronoi cells (polygons) are colored red for CK positive cells and green for CK negative cells. **(E)** Clipped Voronoi tessellation with nucleus centers colored by CK status (red for CK positive cells, green for CK negative cells) and Voronoi cells colored by final tissue type (stroma in green, tumor in red and “other” in blue). **(F)** CK channel colored from black (no CK) to white (maximum CK) with clipped Voronoi tessellation from C overlaid.

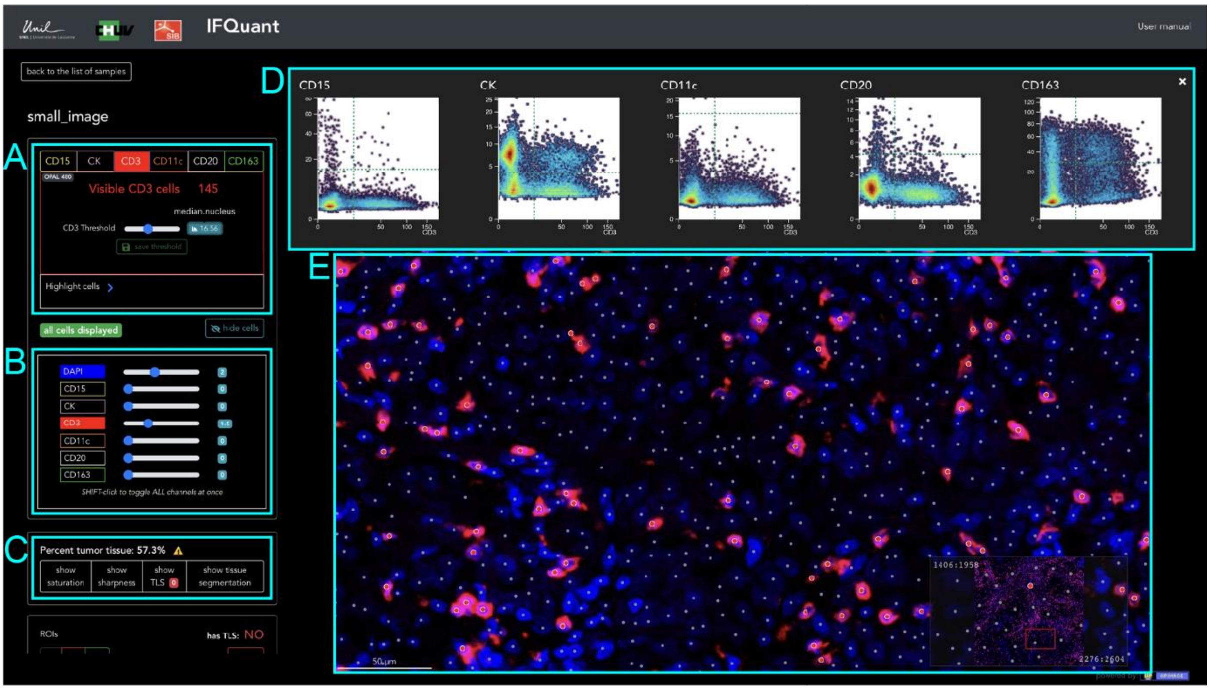

Figure S10: Web application, related to STAR Methods.

A. List of markers. Threshold review/adjustment is done one marker after another. **B.** Enable to combine channels in a composite image. **C.** Display QC, TLS and tissue segmentation masks. **D.** Scatter plots of marker scores for all markers (y-axis) versus the selected marker (x-axis). Marker thresholds are depicted in dotted lines. **E.** Composite image. Positive cells for the selected marker are flagged with a red circle. Negative cells with a smaller grey circle.

Supplemental methods 1

IFQuant

Unmixing

Multiplexed immunofluorescence images contain 8 channels (one channel per fluorophore spectral band and one channel for the autofluorescence). Due to the overlapping emission spectra of the fluorophores, each channel contains a mix of signal from all fluorophores and from autofluorescence.

Let us denote by $H_i^{(n)}$ the fluorescence emitted by fluorophore i at pixel n (H_8 is the autofluorescence) and by $X_i^{(n)}$ the intensity measured in channel i at pixel n (X_8 is the autofluorescence). The measured signal X is approximated as a linear combination of the emitted signal H:

$$X_i^{(n)} = \sum_{j=1}^{8} W_{i,j} H_j^{(n)} + D_i$$

where $\{W_{i,j}\}$ are the elements of the 8×8 non-negative spectrum matrix, $\{D_i\}$ are non-negative offsets.

For a set of N pixels, estimating an approximation of the non-negative spectrum matrix $\{W_{i,j}\}$, offsets $\{D_i\}$ and emitted fluorescence $\{H_j^{(n)}\}$ given a set of measured intensities $\{(X_1^{(n)}, X_2^{(n)}, \cdots, X_8^{(n)}) | n = 1, \cdots, N\}$ can be done using non-negative matrix factorization (NMF) with offset [S1], which is implemented in the R [S2] package NMF [S3]. Additional information can be obtained when using single stained images (one image per fluorophore and an unstained image), for which $H_i^{(n)}$ is expected to be non-zero only for the stained channel and the autofluorescence channel ($i = 8$). This information is used by setting the initial values for $H_i^{(n)}$ to 1 for the stained channel and autofluorescence channel and to 0 for all other channels.

Solving NMF problems is computationally demanding. Instead of considering all pixels from all single stained images at once, 500 pixels are selected in each image so as to span the full range of staining intensities. For each single stained image, pixels are chosen by splitting the range of pixels values from the stained channel into 10 intervals of equal length and for each interval, randomly choosing 50 pixels with stained channel values lying in this interval (or all pixels if less than 50 pixels satisfy this condition). For each selected pixel, a vector of measured intensities $(X_1^{(n)}, X_2^{(n)}, \cdots, X_8^{(n)})$ is obtained by taking the value in each channel, divided by the channel exposure time (in seconds, obtained from the qptiff image metadata). For the unstained image, 500 pixels are chosen at random, and vectors of measured intensities are obtained by normalizing channel values by channel exposure time. For each pixel, a corresponding initial value for the vector of emitted fluorescence $(H_1^{(n)}, H_2^{(n)}, \cdots, H_8^{(n)})$ is obtained by setting all entries to 0 except for the single stained channel and autofluorescence channel, which are set to 1. The NMF with offset algorithm implemented in the R [S2] package NMF [S3] is then run, using as input the 4000 vectors of measured intensities obtained from the 7 single stained image and the unstained image and the corresponding initial values for the emitted fluorescence. To impose the conservation of emitted fluorescence (i.e. columns of the spectrum matrix sum to 1), the resulting spectrum matrix $\{W_{i,j}\}$ and emitted fluorescence $\{H_j^{(n)}\}$ are further normalized as:

$$W'_{i,j} = \frac{W_{i,j}}{\sum_{k=1}^{8} W_{k,j}}$$

and

$$H'^{(n)}_j = H_j^{(n)} \sum_{k=1}^{8} W_{k,j}$$

With this normalization, if $\{W_{i,j}\}$, $\{H_j^{(n)}\}$ and $\{D_i\}$ satisfy the NMF equations, then $\{W'_{i,j}\}$, $\{H'^{(n)}_j\}$ and $\{D_i\}$ also satisfy the NMF equations.

This process of randomly choosing 500 pixels per image and evaluating the normalized spectrum matrix $\{W'_{i,j}\}$ and offsets $\{D_i\}$ is repeated 20 times. The final spectrum matrix and offset vector is taken as the element wise median of $\{W'_{i,j}\}$ and $\{D_i\}$.

The spectrum matrix and offset vector obtained with this procedure can then be used to estimate the contribution of the individual fluorophores to the measured intensities in each channel for new images (unmixing). The fluorescence emitted by fluorophore i at pixel n (H_8 is the autofluorescence) is estimated as

$$H_i^{(n)} = \max \left\{ 0, \sum_{j=1}^{8} \left(W^{-1}\right)_{i,j} \left(X_j^{(n)} - D_j \right) \right\}$$

were W^{-1} is the inverse of matrix W, D_j is the offset for channel j and $X_j^{(n)}$ is the measured intensity obtained by dividing the pixel value in channel j by the channel exposure time (in seconds, obtained from the qptiff image metadata).

In the following, all operations are performed on unmixed images (unmixed on the fly).

Nuclei & cells segmentation

Nuclei segmentation

The nuclei segmentation procedure is heavily inspired by the documentation of the R [S2] package EBImage [S4]. It is done on the unmixed image channel with DAPI nuclear staining (Figure S8A). Nuclei, i.e. regions with local enrichment of DAPI signal, are found by applying an adaptive thresholding with a square window of 15 µm x 15 µm (30 x 30 pixels for an image with 20x pixel resolution) on the blurred DAPI channel (Gaussian kernel with standard deviation of 0.5 µm). The resulting nuclear mask image contains value 1 for pixels in nuclei regions and 0 otherwise (Figure S8B).

To avoid considering low intensity noise as nuclei, regions with blurred DAPI channel (Gaussian kernel with standard deviation of 2.5 µm) below 1 are masked by setting the pixel value of the nuclear mask to 0.

The nuclear mask is cleaned by applying an *opening* morphological operation with a disc of 5 pixels diameter as structuring element and by filling potential holes inside the nuclei.

When too close to each other, nearby nuclei can be merged in the same connected region in the nuclear mask. To split some of these regions and label individual nuclei (using integer cell IDs), the watershed algorithm is applied on the distance map (which contains, for each pixel, the distance to the nearest pixel with value 0) of the nuclear mask (Figure S8C). After this operation, each pixel in the nuclear mask has an integer value corresponding to the cell ID to which it belongs, or 0 for background pixels.

For each nucleus, the nucleus center is obtained as the pixel within the nucleus region which is closest to the center of mass of all pixels in the nucleus region, with mass taken as the intensity of the DAPI channel.

To avoid potential numerical problems, the nuclear mask is cleaned by eroding all nucleus regions by 1 pixel. If a nucleus consists in more than one connected component, the component containing the nucleus center is kept and all other components are considered as background (Figure S8D).

Filtering (out of focus and low dapi)

The sharpness of the DAPI channel is evaluated at each pixel based on the variance of the x and y components of the gradient of the square root transformed DAPI channel. More precisely, let D be the square root transformed DAPI image:

$$D(x, y) = \sqrt{\mathrm{DAPI}(x, y)}$$

for the pixel at position (x, y). The gradient $\nabla D = (S_x, S_y)$ is estimated by convolution with the Sobel operators

$$S_x = \begin{bmatrix} 1 & 0 & -1 \\ 2 & 0 & -2 \\ 1 & 0 & -1 \end{bmatrix} * D$$

and

$$S_y = \begin{bmatrix} 1 & 2 & 1 \\ 0 & 0 & 0 \\ -1 & -2 & -1 \end{bmatrix} * D$$

The variance of each component of the gradient, at position (x, y), is estimated over a circular neighborhood $\Omega(x, y)$ with diameter 20 µm (40 pixels)

$$\mathrm{Var}\left(S_x\right)(x, y) = \frac{1}{N} \sum_{(u,v)\in\Omega(x,y)} S_x(u, v)^2 - \left(\frac{1}{N} \sum_{(u,v)\in\Omega(x,y)} S_x(u, v) \right)^2$$

$$\mathrm{Var}\left(S_y\right)(x, y) = \frac{1}{N} \sum_{(u,v)\in\Omega(x,y)} S_y(u, v)^2 - \left(\frac{1}{N} \sum_{(u,v)\in\Omega(x,y)} S_y(u, v) \right)^2$$

where N is the number of pixels in $\Omega(x, y)$. Finally, the sharpness at position (x, y) is estimated as

$$\mathrm{sharpness}(x, y) = \mathrm{Var}\left(S_x\right)(x, y) + \mathrm{Var}\left(S_y\right)(x, y)$$

The DAPI sharpness of each nucleus is then obtained as the mean sharpness over all pixels in the nucleus region. Similarly, the median DAPI fluorescence of each nucleus is evaluated as the median of the DAPI channel values over all pixels in the nucleus region.

To filter out spurious nuclei detected in low quality regions of the image, all nuclei with DAPI sharpness below 0.5 or median DAPI fluorescence below 0.1 are removed from the list of nuclei.

Cell segmentation

In the absence of a cell membrane staining, the cells regions are approximated by simultaneously extending each nucleus region by up to 5 µm (10 pixels) or until touching a neighboring nucleus region (Voronoi based segmentation [S5]) (Figure S8E).

In addition to nucleus and cell region, we also define the cytoplasm region as the set difference of cell region and nucleus region (Figure S8F).

Per cell fluorescence quantification

For each cell, the distribution of pixel values in each channel (marker) over each region (nucleus, cell, cytoplasm) is summarized by its median, 75-th percentile and 95-th percentile.

In addition, to measure how the marker intensity encircles the nucleus center, the image around the nucleus center is divided into 16 angular sectors. For each region (nucleus, cell, cytoplasm) and each image (marker), the maximum pixel intensity (considering only pixels from the corresponding region) per angular sector is evaluated and summarized by the median. In the following, this quantity is called the encircling median.

Parallelization

To perform all operations, from nucleus segmentation to fluorescence quantification, the image is split in 1600x1600 pixels tiles arranged on a regular grid, with neighboring tiles overlapping by 400 pixels. The computations are performed on each tile independently and the results are subsequently merged.

This part is computationally intensive and is done only once for each image. All subsequent computations are done on the resulting list of summarized properties per cell, such as nucleus center position, mean DAPI sharpness, summary statistics for each region (nucleus, cell, cytoplasm) and each channel.

Cell type assignment

We want to use a simple thresholding approach: assign to each cell one score per channel summarizing the fluorescence in this channel around the cell and classify the cell as positive or negative for this channel depending on whether the score is above or below a predefined threshold.

The simple approach with a unique summary statistic (such as mean or median) over a unique region (e.g. nucleus or cell) for all markers is not optimal for this problem. Indeed, the spatial distribution of fluorescence intensity within the cell depends on the marker (Figure S9A). The fluorescence can be bright and spread over the whole nucleus region (e.g. Ki67, FOXP3) or in the cytoplasm but tightly encircling the nucleus with a "doughnut"-like shape that spills into the nucleus (e.g. CD3, CD4, CD8). For other markers (e.g. CK), the fluorescence is more diffuse and fills the space outside of the nucleus. Alternatively, the fluorescence can be limited to bright small dots inside or close to the nucleus (e.g. GB). More problematic markers (e.g. CD163, CD11c or PDL1), are expressed in the cytoplasm but the fluorescence can be far away from the nucleus, not necessarily encircling the nucleus. For some markers, such as CD15, the spatial distribution of fluorescence can depend on the context: when coexpressed with CK, CD15 has a low and diffuse cytoplasmic fluorescence that overlaps CK staining, while in the absence of CK, CD15 fluorescence tends to be stronger and tightly surrounding the nucleus.

Another complication is the presence of residual autofluorescence and fluorescence from other fluorophores which remains after unmixing. This is particularly problematic for markers with low intensity staining, as it can be difficult to distinguish positive signal from residual background. Spatial distribution of fluorescence can help distinguish the targeted marker from residual fluorescence.

To take into account information on the specific spatial distribution of fluorescence for each marker, we use one score per marker, defined as the combination of a region (nucleus, cytoplasm, or cell) and a summary statistic (median, 75-th percentile, 95-th percentile or encircling median) over this region. This score is empirically chosen to be as specific as possible for the expected spatial distribution of fluorescence of the targeted marker. Let us consider a few examples to illustrate this idea (see Figure S9B). To detect small bright dots inside the nucleus (e.g. GB), we can use the 95-th percentile over the nucleus region, as this score will be dominated by the top 5% pixels in the nucleus region with highest fluorescence. If the fluorescence should fill the nucleus (e.g. Ki67, FOXP3), a possible choice of score is the median over the nucleus region, as it ensures that half of the pixels in the nucleus have a fluorescence above this score. For markers with a bright doughnut-like distribution of fluorescence

encircling the nucleus and spilling into the nucleus, possibly filling the nucleus (e.g. CD3, CD4, CD8), the median over the nucleus can be a good choice, as it enforce that the fluorescence is close enough to the nucleus to significantly fill the nucleus region. Similarly, the median over the cytoplasm can be used for diffuse markers filling the space outside the nucleus (e.g. CK). The encircling median over the cell or cytoplasm is appropriate when the fluorescence is expected to encircle the nucleus (e.g. CD163, CD11c), at least partially. It is also a good compromise for markers such as CD15 that can have a fluorescence that is either a diffuse over the cytoplasmic region or more present around or into the nucleus region as it ensures that half of the directions around the nucleus center have a fluorescence above this score.

For this study, the following settings were used:

Marker	Statistic	Region
CD15	encircling median	cell
CK	median	cell
CD3	median	nucleus
CD11c	encircling median	cell
CD20	75-th percentile	nucleus
CD163	encircling median	cytoplasm

Given a set of thresholds, estimated empirically for each marker, each cell is assigned to a raw phenotype, by classifying the cell as positive or negative for each marker depending on whether the score for this maker is above or below the threshold. A phenotype key can then be used to map these raw phenotypes (e.g. CD15-CK-CD3-CD11c-CD20+CD163-) to final phenotypes (e.g. B cell).

Tissue segmentation

CK threshold estimation

For each cell, the CK score is chosen as the median of the pixel intensities in the CK channel over the cell region. For an image with a balanced distribution of stroma and tumor, the distribution of CK score is expected to be bimodal, with a low CK population corresponding to stromal cells and a high CK population corresponding to tumor cells. In general, though, an image might be dominated by tumor (or stroma) cells, possibly resulting in a unimodal distribution of CK score. To increase the chances of finding a bimodal distribution of CK scores, the image is split in 1500x1500 pixels tiles arranged on a regular grid, with neighboring tiles overlapping by 750 pixels. For each tile, to avoid using cells detected in low-quality regions, only cells with mean DAPI sharpness above 5 are considered. If the tile has more than 500 remaining cells, the distribution of asinh transformed CK scores for all cells in the tile is approximated using a gaussian kernel density estimation, with bandwidth $w = \max\{m/20, 0.01\}$ (m is the maximum asinh transformed CK score in the tile). If the distribution is bimodal, the position of the local minimum is stored, otherwise the tile is ignored. The final threshold for the CK score is taken as the median of all CK scores corresponding to local minima.

Segmentation

Information on cell size and local neighborhood is estimated using a Delaunay triangulation for the set of nucleus centers (Figure S9C). The corresponding Voronoi tessellation is also created to approximate cell regions. To impose a limit on cells sizes, each cell of the Voronoi tessellation is clipped to a maximum distance to nucleus center of 15 µm (30 pixels, Figure S9D). The area of the clipped Voronoi cell is used as an approximation of the cell area.

A first "naive" tissue segmentation is done by assigning all cells with CK score above the CK threshold to tumor tissue type and all other cells to stroma tissue type (Figure S9D). A local neighborhood

graph G is created from the Delaunay triangulation, using nuclei centers as vertices, and keeping only edges with length below 40 µm (81 pixels). Connected groups of stroma (respectively tumor) cells are found by searching for connected components in the subgraph of G induced by the set of nuclei with stroma (respectively tumor) tissue type. Groups of stroma (respectively tumor) cells with up to 5 cells connected to a group of tumor (respectively stroma) cells than with more than 10 cells are selected and their tissue type is changed to tumor (respectively stroma). Groups of cells with up to 5 cells with same tissue type, which are not connected to any other cells are assigned to tissue type “other” (Figure S9E,F).

After tissue segmentation, cell areas and tissue types are added to the list of cell properties.

TLS

IFQuant uses patches of CD20+CK- cells as a proxy for tertiary lymphoid structures (TLS).

First, the local density of CD20+CK- cells (cells with CD20 score above CD20 threshold and CK score below CK threshold) is estimated, for each CD20+CK- cell, as $k/(\pi r^2)$, where r is the distance to the k-th nearest CD20+CK- cell and k=4 (kNN density estimator [S6]).

CD20+CK- cells with a local density of CD20+CK- cells above 2000 cells/mm^2 are then selected and patches of CD20+CK- cells are found as connected components of the alpha shape [S7] (with a disc of radius 20 µm) for the set of selected CD20+CK- cells (using the position of the nucleus center). Patches containing less than 40 selected cells are excluded.

Finally, all cells with nucleus center lying inside the region delimited by the external boundary of a patch are assigned to this patch.

Visualization and parameters adjustment

The output of cell and tissue segmentation described above consists in a list of summarized properties per cell: nucleus center position, mean DAPI sharpness, tissue type, cell area and one score per marker.

In addition, several images are precomputed and stored in tiled pyramidal tiff format: a saturation mask showing for each pixel whether it saturates in any channel, a QC mask based on DAPI sharpness, an unmixed image (one band per channel), a set of tissue type masks for various CK thresholds and a TLS mask. Note that the precomputed unmixed images are only used for visualization. Analyses are done on images unmixed on the fly.

These results are displayed in a web application (Figure S10). This application allows to visualize the full resolution image (with zooming and paning functionalities), with custom combinations of the channel intensities in false colors. It can overlay the QC (saturation, sharpness) and TLS masks. Two features help adjusting the threshold for each marker: highlighting with a red circle positive cells in the image and displaying the thresholds on scatter plots of marker scores (for all pairs of markers). The application also allows to manualy draw regions to exclude and regions of interest (ROIs). Summary statistics are displayed and reports (PDF and XLSX) can be dowloaded.

The application is using the IIPImage image server to combine the different channels and stream image tiles to the image viewer (based on the OpenSeaDragon javascript library). The frontend interface is developped with the VueJS (version 2) framework. The backend is developped in PHP with the Slim framework. To speed up the application, the tab-separated table generated in the report is imported and indexed in a SQLite database.

Report

Once all settings are validated a report is created. This report presents all settings used for the analysis, QC images as well as tables summarizing number of cells, densities, and areas for the whole image

and per region of interest, aggregated by tissue type and by phenotype. All tables are also saved in xlsx format. Finally, a table in tab-separated format contains a list of properties for each cell: nucleus center position, cell area, one score per marker, one normalized score per marker (score divided by threshold), raw phenotype (specifying for each marker whether its score is above or below threshold), whether the cell is in a TLS or not, and whether the cell is in a ROI or not.

Implementation

- Bio-formats command line tools v6.6.0 [S8] (https://www.openmicroscopy.org/bio-formats/downloads/).
- libvips v8.9.1 [S9] (https://libvips.github.io/libvips/).
- R v4.0.4 [S2] (https://www.R-project.org) with the packages:
  - EBImage v4.32.0 [S4] (http://bioconductor.org/packages/EBImage).
  - NMF v0.23.0 [S3] (https://CRAN.R-project.org/package=NMF).
  - geometry v0.4.5 (https://CRAN.R-project.org/package=geometry).
  - igraph v1.2.6 [S10] (https://CRAN.R-project.org/package=igraph).
  - writexl v1.3.1 (https://CRAN.R-project.org/package=writexl).
  - rmarkdown v2.7 [S11] [S12] (https://rmarkdown.rstudio.com).
  - data.table v1.14.0 (https://CRAN.R-project.org/package=data.table).
  - ggplot2 v3.3.3 [S13] (https://ggplot2.tidyverse.org).
  - R.utils v2.10.1 (https://CRAN.R-project.org/package=R.utils).
  - gridExtra v2.3 (https://CRAN.R-project.org/package=gridExtra).
  - viridis v0.5.1 (https://CRAN.R-project.org/package=viridis).
  - gplots v3.1.1 (https://CRAN.R-project.org/package=gplots).
  - KernSmooth v2.23-18 (https://CRAN.R-project.org/package=KernSmooth).
  - RColorBrewer v1.1-2 (https://CRAN.R-project.org/package=RColorBrewer).
  - RANN v2.6.1 (https://CRAN.R-project.org/package=RANN).
  - sp v1.4-5 (https://CRAN.R-project.org/package=sp).
- IIPimage v1.2 (https://iipimage.sourceforge.io/).
- VueJS v2.7.14 (https://vuejs.org/).
- OpenSeadragon v3.1.0 (https://openseadragon.github.io/).
- BootstrapVue v2.23.1 (https://bootstrap-vue.org/).
- FabricJS v4.6.0 (http://fabricjs.com/).
- PHP v8.0 (https://www.php.net/).
- Slim v3.12.4 (https://www.slimframework.com/).

Supplemental methods 2

Gating strategy to label cell types for IMMUcan panel 1

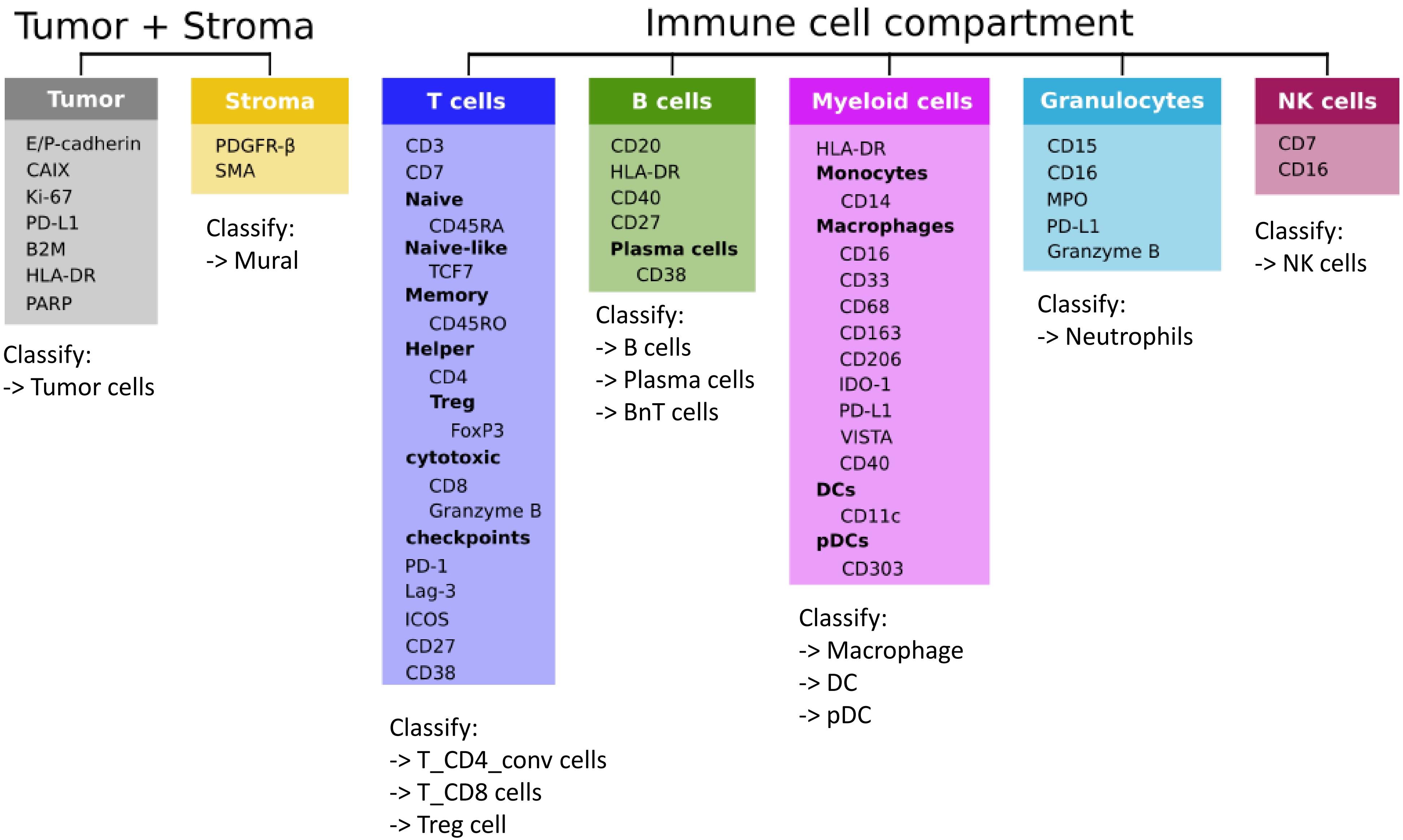
Tumor + Stroma
Immune cell compartment
Tumor
E/P-cadherin
CAIX
Ki-67
PD-L1
B2M
HLA-DR
PARP
Classify:
-> Tumor cells
Stroma
PDGFR-β
SMA
Classify:
-> Mural
T cells
CD3
CD7
Naive
CD45RA
Naive-like
TCF7
Memory
CD45RO
Helper
CD4
Treg
FoxP3
cytotoxic
CD8
Granzyme B
checkpoints
PD-1
Lag-3
ICOS
CD27
CD38
Classify:
-> T_CD4_conv cells
-> T_CD8 cells
-> Treg cell
B cells
CD20
HLA-DR
CD40
CD27
Plasma cells
CD38
Classify:
-> B cells
-> Plasma cells
-> BnT cells
Myeloid cells
HLA-DR
Monocytes
CD14
Macrophages
CD16
CD33
CD68
CD163
CD206
IDO-1
PD-L1
VISTA
CD40
DCs
CD11c
pDCs
CD303
Classify:
-> Macrophage
-> DC
-> pDC
Granulocytes
CD15
CD16
MPO
PD-L1
Granzyme B
Classify:
-> Neutrophils
NK cells
CD7
CD16
Classify:
-> NK cells

General notes on gating

Positive gating

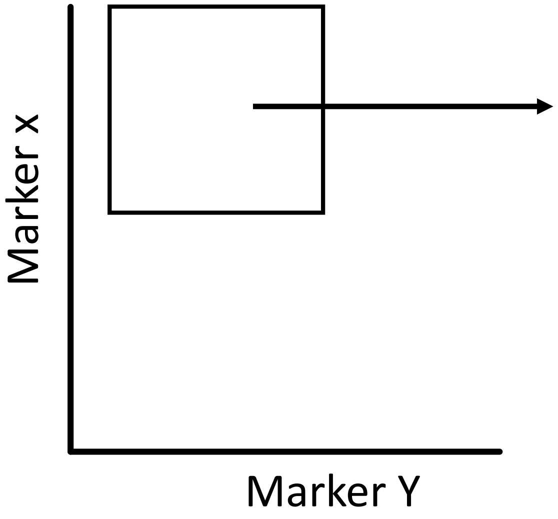

In a depiction like this cells were selected with positive expression for marker X and negative for marker Y. Cells from this gate were then passed to the next selection.

Negative gating

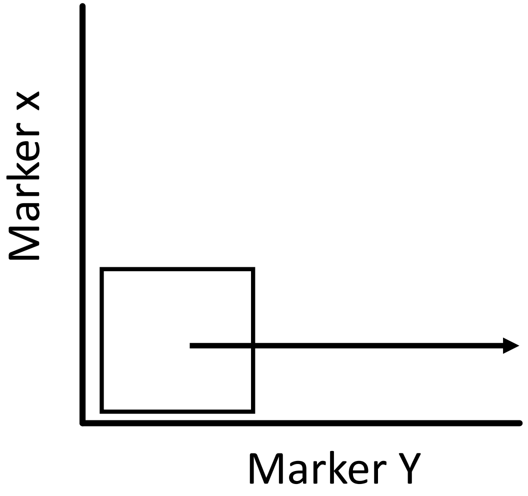

For negative selection of markers cells with no expression are selected.

Final gate

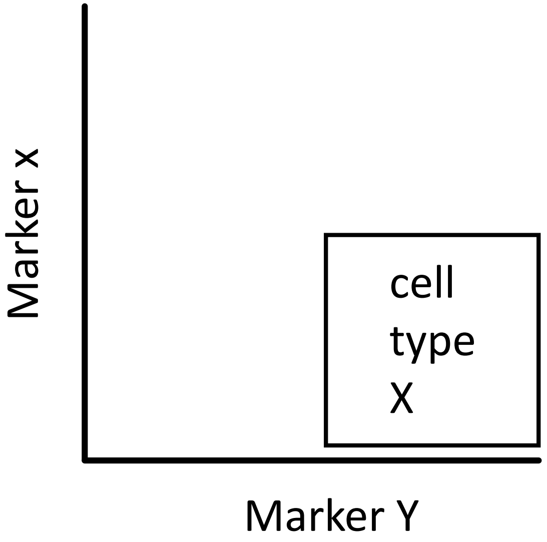

In the final gate the respective cell type is labelled

Tumor cell definition

Note:
tumors can often express HLADR. therefore the dataset for labelling should include HLADR+Ead+ cells. The HLADR gate is only for visual control.

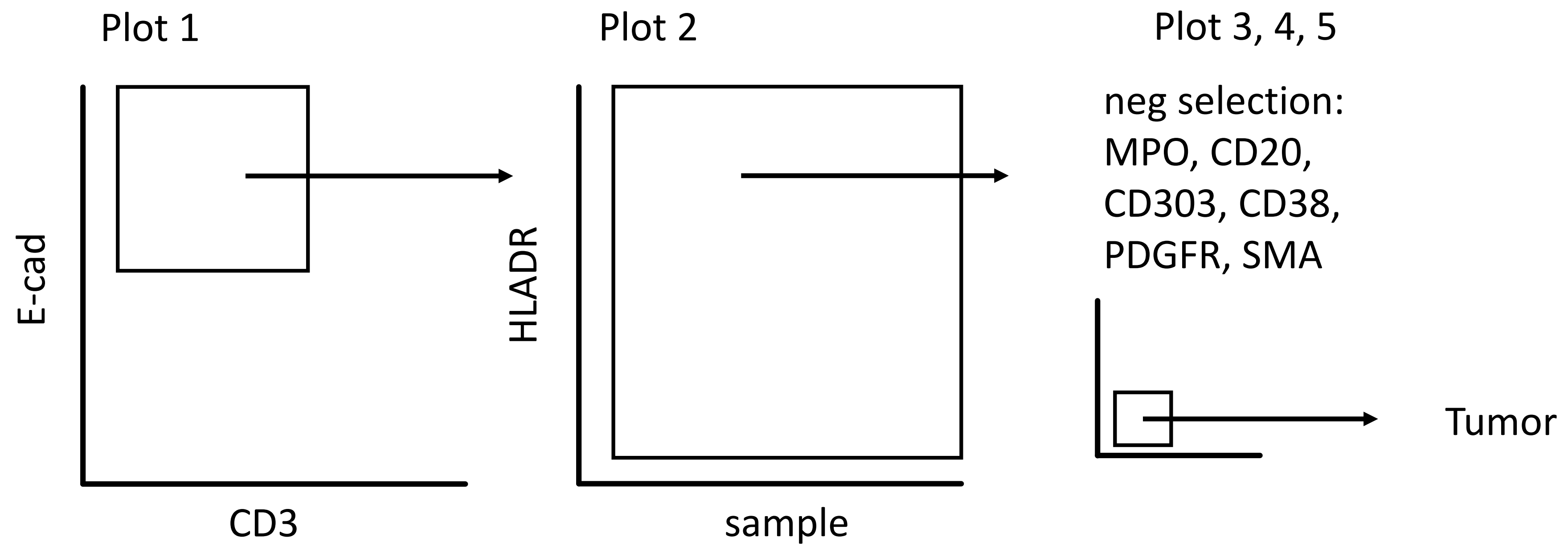

Note:
In RCC samples the tumor cells are often E-cad$^-$ but Carbonic anhydrase$^+$

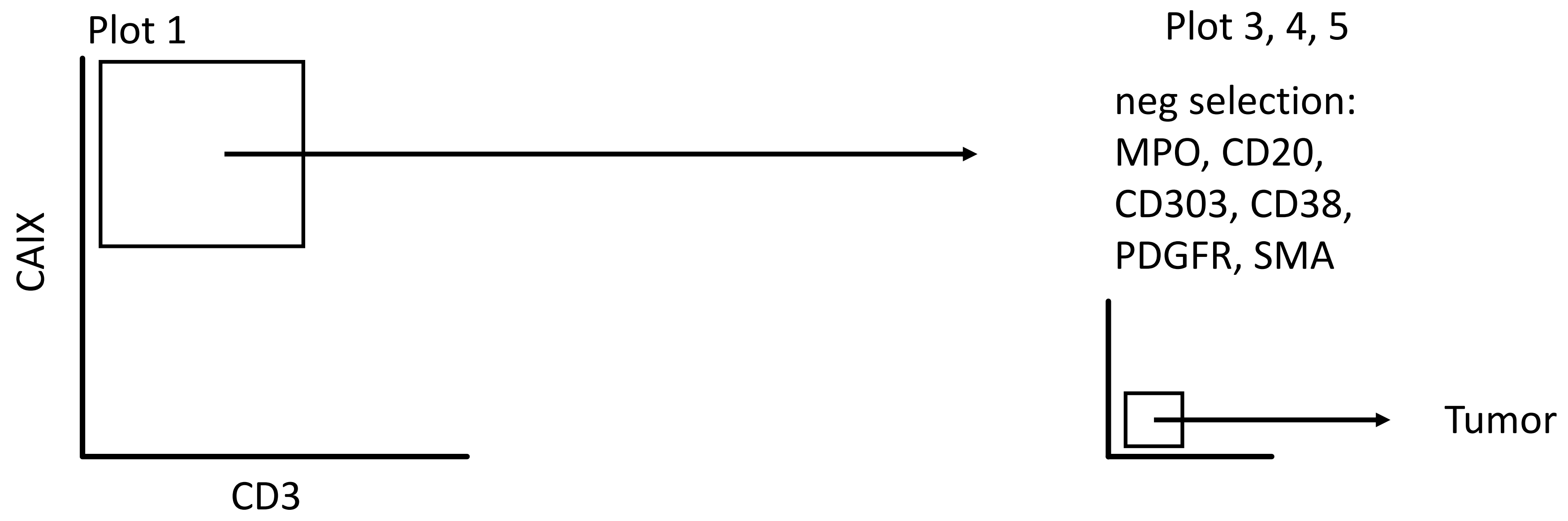

Myeloid cell definition

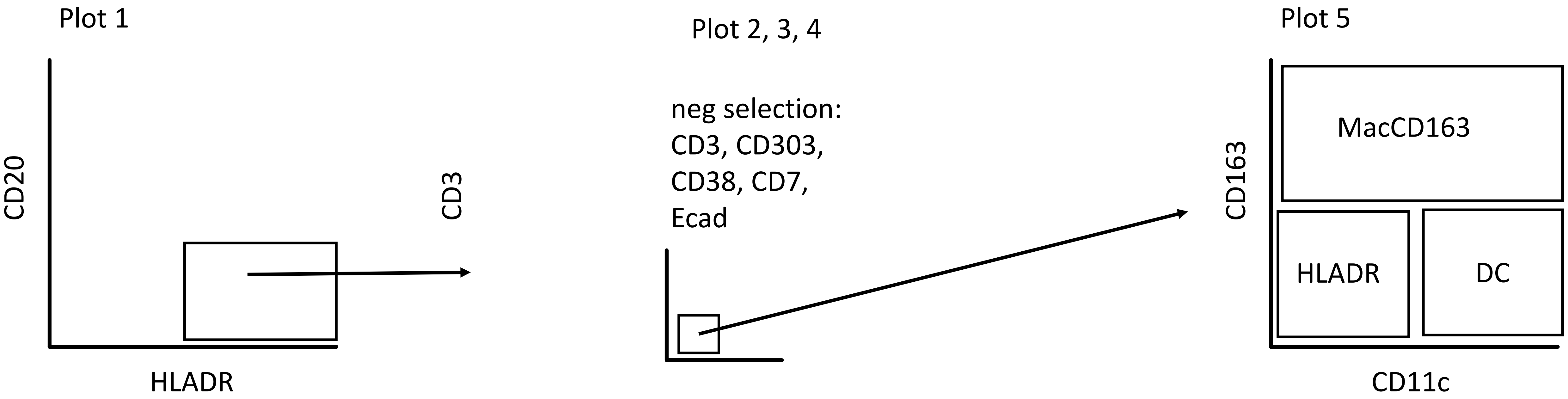

Note:
In samples with TLS the negative gates for CD3 and CD20 should not be used in order to obtain DCs in the tight mesh of B and T cells.

Plasmacytoid DC definition

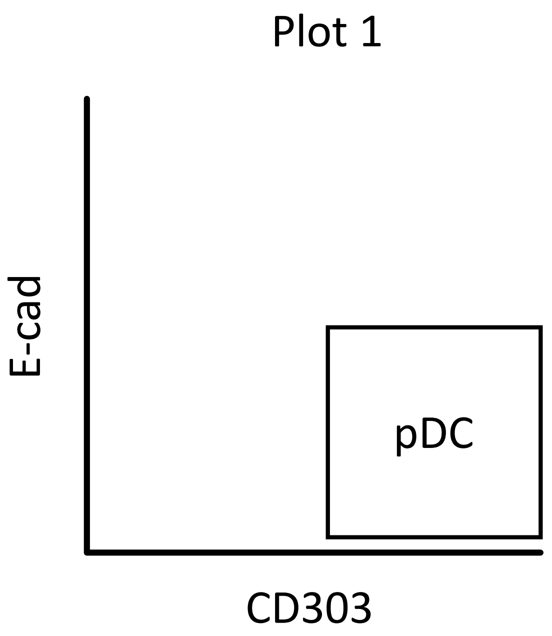

Neutrophil definition

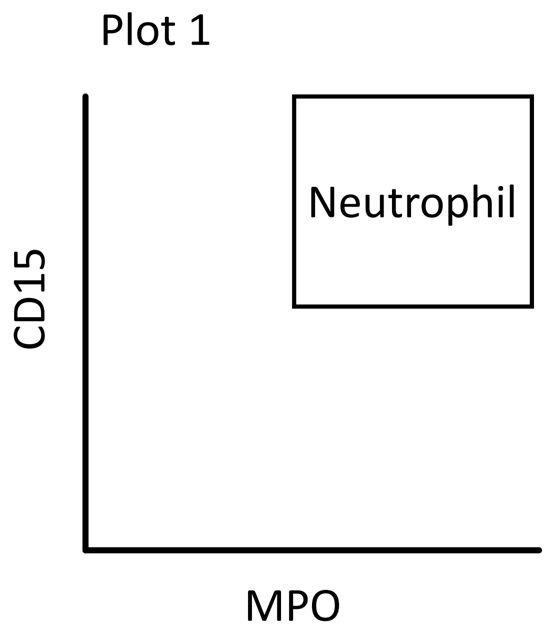

Plasma cell definition

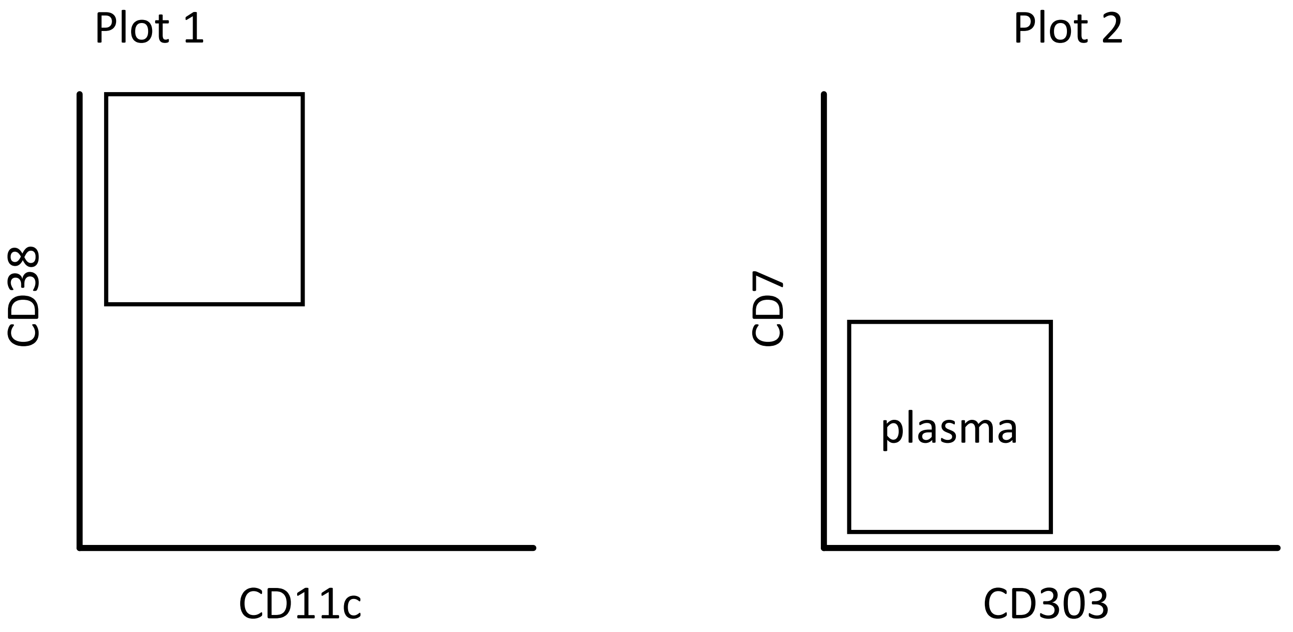

Note:
Use the negative gate to actually not loose any of the labelled pDCs, DCs and later on NK cells

Mural cell definition

Plot 1, 2, 3, 4

neg selection:
CD3, Ecad, CD20, CD38,
MPO, CD303, HLADR,
CD7

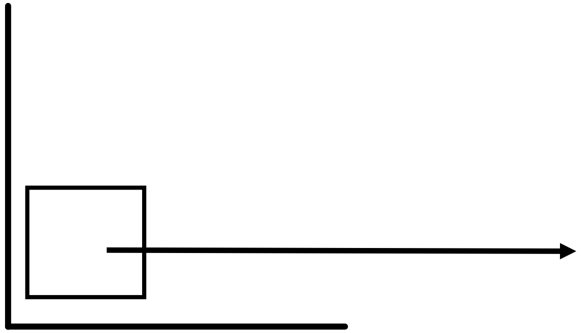

Plot 5

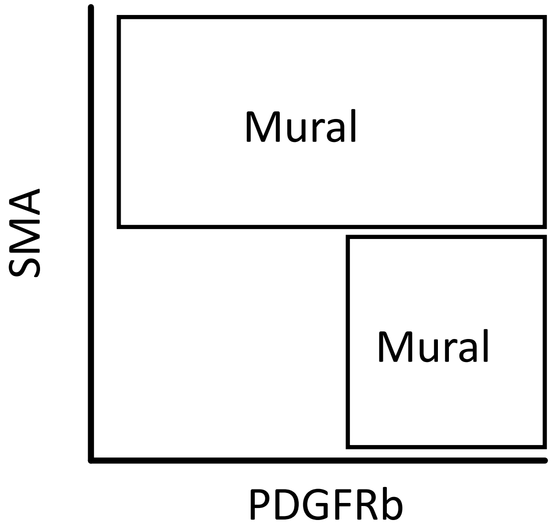

B cell and intermixed B and T cell (BnT) definition

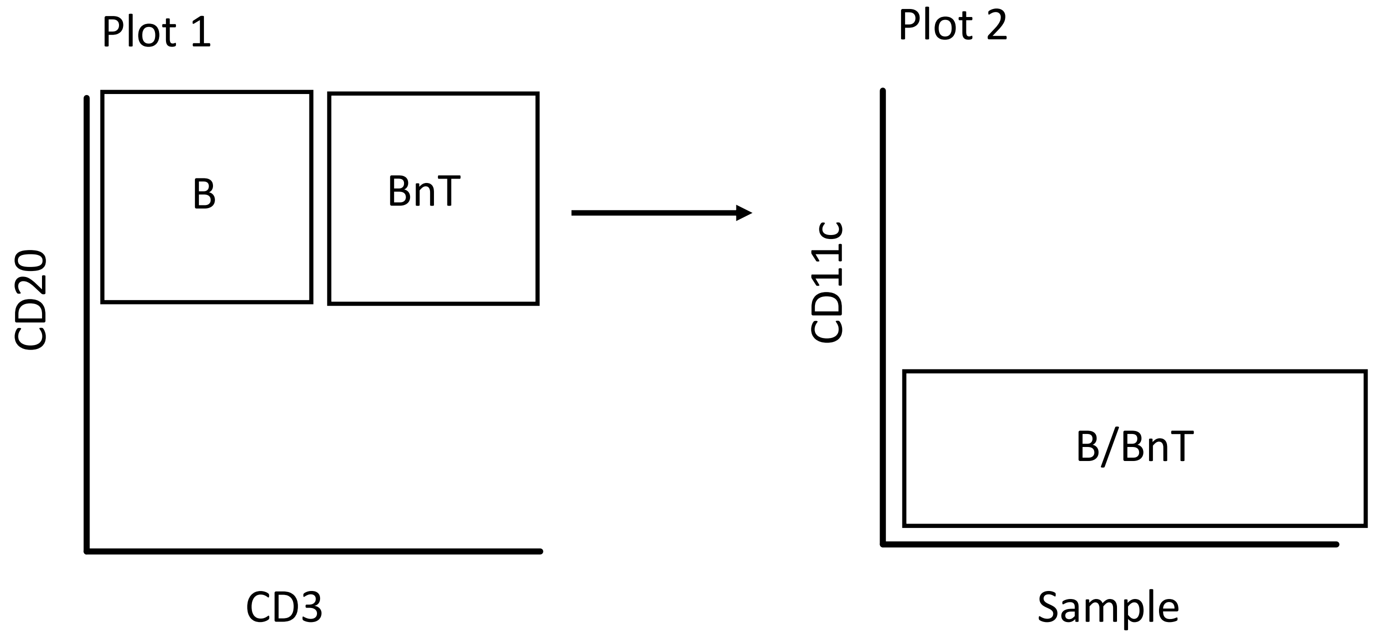

To account for DCs inside of B cell patches or TLS we specifically excluded B or BnT cells with high CD11c expression from the final gates

NK cell definition

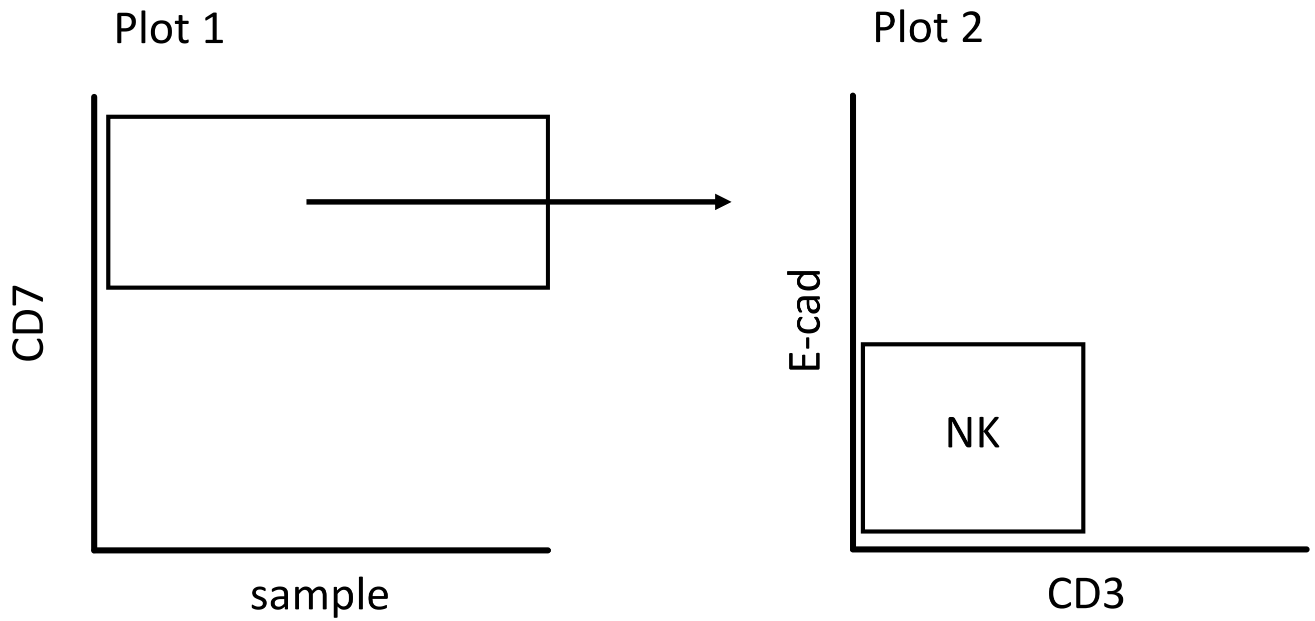

T cell definition

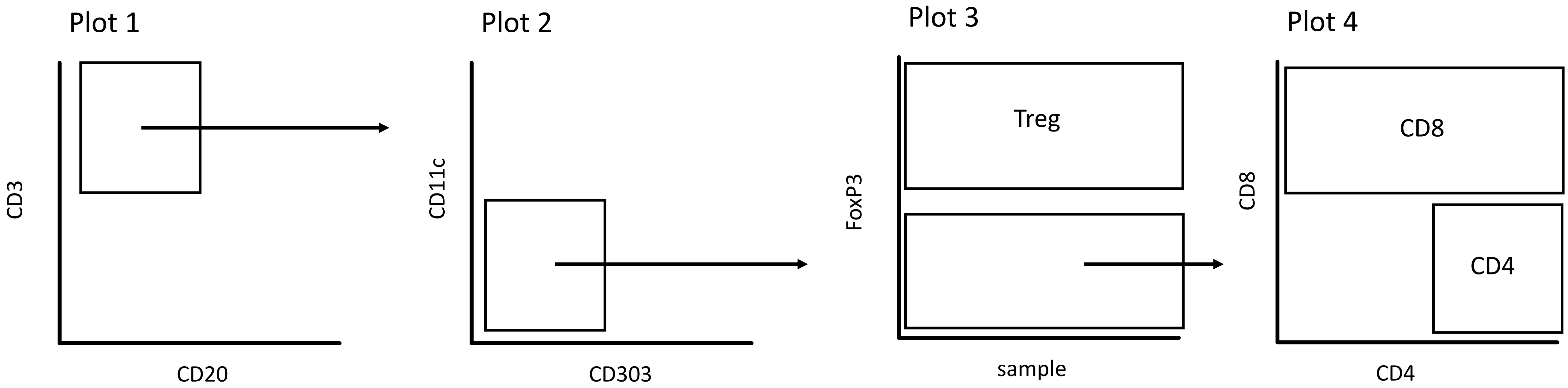

Supplemental references

[S1] Badea, L. (2008). Extracting gene expression profiles common to colon and pancreatic adenocarcinoma using simultaneous nonnegative matrix factorization. Pacific Symposium on Biocomputing 13, 267-278.

[S2] R Core Team (2021). R: A language and environment for statistical computing (R Foundation for Statistical Computing). https://www.R-project.org/.

[S3] Gaujoux, R., and Seoighe, C. (2010). A flexible R package for nonnegative matrix factorization. BMC Bioinformatics 11, 367. 10.1186/1471-2105-11-367.

[S4] Pau, G., Fuchs, F., Sklyar, O., Boutros, M., and Huber, W. (2010). EBImage - an R package for image processing with applications to cellular phenotypes. Bioinformatics 26, 979-981. 10.1093/bioinformatics/btq046.

[S5] Jones, T.R., Carpenter, A., and Golland, P. (2005). Voronoi-Based Segmentation of Cells on Image Manifolds. In Computer Vision for Biomedical Image Applications (CVBIA), Liu, Y., Jiang, T., Zhang, C., eds. (Springer), pp. 535-543. 10.1007/11569541_54.

[S6] Loftsgaarden, D.O., and Quesenberry, C.P. (1965). A Nonparametric Estimate of a Multivariate Density Function. The Annals of Mathematical Statistics 36, 1049–1051. 10.1214/aoms/1177700079.

[S7] Edelsbrunner, H., Kirkpatrick, D., and Seidel, R. (1983). On the shape of a set of points in the plane. IEEE Transactions on Information Theory 29, 551–559. 10.1109/TIT.1983.1056714.

[S8] Linkert, M., Rueden, C.T., Allan, C., Burel, J.-M., Moore, W., Patterson, A., Loranger, B., Moore, J., Neves, C., MacDonald, D., et al. (2010). Metadata matters: Access to image data in the real world. Journal of Cell Biology 189, 777–782. 10.1083/jcb.201004104.

[S9] Martinez, K., and Cupitt, J. (2005). VIPS - a highly tuned image processing software architecture. In IEEE International Conference on Image Processing 2, pp. 574-577. 10.1109/ICIP.2005.1530120.

[S10] Csardi, G., and Nepusz, T. (2006). The igraph software package for complex network research. InterJournal, Complex Systems, 1695, 1-9.

[S11] Xie, Y., Allaire, J.J., and Grolemund, G. (2018). R Markdown: The Definitive Guide (Chapman and Hall/CRC).

[S12] Xie, Y., Dervieux, C., and Riederer, E. (2020). R Markdown Cookbook (Chapman and Hall/CRC).

[S13] Wickham, H. (2016). ggplot2: Elegant Graphics for Data Analysis (Springer-Verlag).
